# Prevalence, risk factors and genetic characterisation of extended-spectrum beta-lactamase and carbapenemase-producing Enterobacteriaceae (ESBL-E and CPE): a community-based cross-sectional study, the Netherlands, 2014 to 2016

**DOI:** 10.2807/1560-7917.ES.2019.24.41.1800594

**Published:** 2019-10-10

**Authors:** Gerrita van den Bunt, Wilfrid van Pelt, Laura Hidalgo, Jelle Scharringa, Sabine C. de Greeff, Anita C. Schürch, Lapo Mughini-Gras, Marc J.M. Bonten, Ad C. Fluit

**Affiliations:** 1 Julius Center for Health Sciences and Primary Care, University Medical Center Utrecht (UMCU), Utrecht, the Netherlands; 2 Centre for Infectious Disease Control, National Institute for Public Health and the Environment (RIVM), Bilthoven, the Netherlands; 3 Department of Medical Microbiology, University Medical Center Utrecht (UMCU), Utrecht, the Netherlands; 4 Institute for Risk Assessment Sciences (IRAS), Faculty of Veterinary Medicine, Utrecht University, Utrecht, the Netherlands

**Keywords:** Extended Spectrum Beta-Lactamase (ESBL), prevalence, risk factors, genetic characterization, general community

## Abstract

**Background:**

The epidemiology of carriage of extended-spectrum beta-lactamase-producing (ESBL-E) and carbapenemase-producing Enterobacteriaceae (CPE) in the general population is unknown.

**Aim:**

In this observational study, the prevalence and risk factors for intestinal ESBL-E and CPE carriage in the Dutch general population were determined. ESBL-E were characterised.

**Methods:**

From 2014 to 2016, ca 2,000 residents were invited monthly to complete a questionnaire and provide a faecal sample, which was tested for ESBL-E. The first 1,758 samples were also tested for CPE. Risk factors for ESBL-E carriage were identified by multivariable logistic regression analysis. ESBL-E isolates underwent whole genome sequencing.

**Results:**

Of 47,957 individuals invited, 4,177 (8.7%) completed the questionnaire and provided a faecal sample. ESBL-E were detected in 186 (4.5%) individuals, resulting in an adjusted prevalence of 5.0% (95% confidence interval (CI):3.4–6.6%). Risk factors were: born outside the Netherlands (odds ratio (OR): 1.99; 95% CI: 1.16−4.54), eating in restaurants > 20 times/year (OR: 1.70; 95% CI: 1.04−2.76), antibiotic use < 6 months ago (OR: 2.05; 95% CI: 1.05−4.03), swimming in sea/ocean < 12 months ago (OR: 1.63; 95% CI: 1.11−2.39), travelling to Africa (OR: 3.03; 95% CI: 1.23−7.46) or Asia (OR: 2.00; 95% CI: 1.02−3.90) < 12 months ago, and not changing kitchen towels daily (OR: 2.19; 95% CI: 1.24−3.87). The last had the largest population attributable risk (PAR) (47.5%). Eighty-four of 189 (44.4%) ESBL-E isolates carried *bla*_CTX-M-15_. *Escherichia coli* isolates belonged to 70 different sequence types (ST)s, of which ST131 (42/178 isolates; 23.6%) was most prevalent. Associations were observed between IncFIA plasmids and ST131 and *bla*_CTX-M-27_, and between IncI1 and ST88 and *bla*_CTX-M-1_. No CPE were detected.

**Conclusions:**

The prevalence of ESBL-E carriage in the Netherlands’ community-dwelling population is 5.0%. Identified risk factors were mostly travelling (particularly to Asia and Africa) and kitchen hygiene. CPE were not detected.

## Introduction

Extended-spectrum beta-lactamase producing Enterobacteriaceae (ESBL-E) are present in different niches [1-4], providing multiple opportunities for transmission. In the Netherlands, the proportion of ESBL-producing bacteria among those causing invasive infections increased to around 7% in 2015, and has stabilised ever since [5]. Although some assessments of prevalence of ESBL-E carriage in the community have been made, all these studies focused on specific groups, such as farmers [6], travellers [7,8], general practitioner’s (GP) patients [9], households with young children [10], or those living in either densely populated urban areas [11] or in rural areas [12,13]. Reported prevalence of ESBL-E carriage varied and was 10.6% among patients visiting the GP [9], 8.6% among persons living in an urban area [11], 8.6% among people before travel [7], 4.5–5.1% among individuals living in rural areas [12,13], and 4.0% in households with young children [10]. Yet, none of the aforementioned study populations reflected the true general population. A better understanding of the prevalence of and risk factors for carriage with ESBL-E in the general population might enhance our capacity to detect carriers entering the healthcare system. Moreover, identification of risk factors for carriage with specific ESBL-E genotypes, plasmids or resistance genes might allow more targeted screening strategies.

This study aimed to determine the prevalence and risk factors for ESBL-E carriage in the Dutch general population. We also genetically characterised ESBL-E, from faecal samples of volunteers in the Dutch community, including ESBL genes and ESBL-carrying plasmids.

## Methods

### Study design, setting and data collection

A monthly-repeated cross-sectional study among residents in the Netherlands was performed from November 2014 to November 2016. Each month, a random sample stratified by county and degree of urbanisation (≥ 2,500; 1,500–2,500, 1,000–1,500, 500–1,000, < 500 addresses per km^2^) of ca 2,000 residents, including all ages, was drawn from Dutch municipal registries covering the country’s population (ca 17 million inhabitants). One person per household was invited by regular mail to complete a web-based questionnaire, which was developed based on previous studies [10,14,15], with additional questions about education, medicine use, hospitalisation, travelling, and contact with animals.

Upon completion of the questionnaire with 120 questions, all participants were asked to provide a faecal sample, and if willing, a stool sample collection kit was provided accompanied with a second short web-based questionnaire with 24 questions similar to the first one, but referring to the four-week period before faecal sample collection. The English translation of the used questionnaires, which were in Dutch are provided in Supplement S1 and S2.

### Extended-spectrum beta-lactamase producing Enterobacteriaceae detection

Samples were cultured overnight at 37 °C on MacConkey agar with 1 mg/L cefotaxime. In addition, all samples were cultured overnight at 37 °C in enrichment broth consisting of 2 mL of lysogeny broth (LB) with 1 mg/L cefotaxime. When growth was observed on the MacConkey agar, up to five colonies with different morphologies were selected and re-cultured on MacConkey agar with 1 mg/L cefotaxime. The broths enriched with 2 mL of LB with 1 mg/L cefotaxime were discarded in these cases. In the absence of growth on the MacConkey plate with 1 mg/L of cefotaxime the enrichment broth was cultured on MacConkey agar with 1 mg/mL cefotaxime. When growth was present on the agar plate agar up to five colonies with different morphologies were selected and re-cultured on MacConkey agar with 1 mg/L cefotaxime. The isolates were stored in LB with 30% glycerol at -80 °C until further use. Isolates were speciated by MALDI-TOF (Bruker, Bremen, Germany). Only isolates belonging to *Escherichia coli*, *Klebsiella pneumoniae*, and the *Enterobacter cloacae* complex were further studied.

Bacterial DNA was first purified using the MO BIO Ultra Clean Microbial DNA isolation kit (Qiagen, Carlsbad, California (CA)) and thereafter checked by specific PCR for the presence of genes encoding CTX-M group 1 and 9 ESBLs as these are the most common. Isolates negative in the PCR were tested for the presence of other ESBL-encoding genes by the Check MDR CT-101 microarray (Check-points, Wageningen, the Netherlands). ESBL-encoding genes were identified by specific PCRs and subsequent sequencing of the amplification products by conventional ABI sequencing technology (ThermoFisher, Waltham, Massachusetts (MA)). PCR-based replicon typing (pBRT) was performed to identify the plasmid type that encoded the ESBL [16].

For whole genome sequence analysis, the same bacterial DNA was used to prepare a library for sequencing with the MiSeq or Nextseq (Illumina, San Diego, CA) platforms, using the NexteraXT library prep kit (Illumina). Contigs were assembled with SPAdes genome assembler v.3.6.2 and contigs larger than 500 bp and with at least 10x coverage were further analysed. The assembled contigs were analysed for the presence of resistance genes, genes encoding virulence factors, replicon types and multilocus sequence type (ST) by ResFinder, VirulenceFinder, PlasmidFinder, and multilocus sequence typing (MLST) available from the Center for Genomic Epidemiology (DTU, Copenhagen, Denmark) [17-20].

### Carbapenemase-producing Enterobacteriaceae (CPE) detection

A part of each faecal sample was stored in guanidine isothiocyanate buffer, and the first 1,758 faecal samples underwent real-time PCR with the Check-Direct CPE kit (Check-points, Wageningen, the Netherlands) on a LC480 (Roche, Almere, the Netherlands) for the presence of *K. pneumoniae* carbapenemase (KPC), New Delhi metallo-beta-lactamase (NDM), oxacillinase (OXA)-48-group, Verona integron-encoded metallo-beta-lactamase (VIM) and imipenemase (IMP) coding genes. DNA purification was performed with a MagnaPure MP96 (Roche, Almere, the Netherlands).

### Data analysis

The overall prevalence of carriage of ESBL-producing bacteria was calculated and weighted proportionally to the Dutch reference population, as recorded on 31 December 2016, taking age (0–18, 19–40, 41–60, > 60 years), ethnicity (native or not native Dutch (at least one parent not born in the Netherlands)) and degree of urbanisation of the place of residence (≥ 2,500, 1,500–2,500, 1,000–1,500, 500–1,000, < 500 addresses per km^2^) into account [21].

A total of 79 putative risk factors were assessed by chi-squared or Fisher exact tests and a Benjamini and Hochberg correction for multiple testing was applied to p values in order to control the false discovery rate (FDR) [22]. A FDR of 0.15 was used. Only variables significant (p ≤ 0.05) after the Benjamini and Hochberg correction were included in univariate logistic regression, as well as risk factors described in literature. Variables with a p ≤ 0.10 were selected for inclusion in a multivariable model built in backward stepwise fashion, with variables with p < 0.05 being retained in the final model. In the multivariable analyses, we controlled for age and season, and tested for all possible interactions. If the covariates changed ≥ 10%, the variable remained in the model. Missing values (0–25%) were imputed using chained equations, imputing and pooling estimates from 10 random datasets [23]. A sensitivity analysis was performed based on the imputed data and compared with complete case analyses.

For each individual, we calculated the predicted probability of ESBL carriage, based on the risk factors identified from the final multivariable logistic regression model using the predict function of the ‘stats’ package in R (R Foundation for Statistical Computing, Vienna, Austria). Since the threshold to classify an individual as ESBL positive based on the predicted probability is arbitrary, we decided to calculate a positive predictive value (PPV) and negative predictive value (NPV) for every threshold between 0 and 1, with steps of 0.001 to determine when the maximum PPV and NPV was reached.

The population attributable risk (PAR), which is the proportion of ESBL carriers that would be prevented following elimination of the exposure, assuming the exposure is causal, was calculated by using the adjusted odds ratios (OR) from the final multivariable logistic regression model for the variables significantly associated with ESBL carriage, and the prevalence of exposure in cases. Similarly, the 95% confidence intervals (95% CI) of the PAR values were calculated based on the 95% CI of the OR [24].

Weighted prevalence was calculated using SAS version 9.1.3 (SAS Institute Inc., Cary, North Carolina (NC)), and R version 3.2.2 was used for all other statistical analyses.

Power calculations were predisposed for ‘exposed’ and ‘non-exposed’ to the potential risk factor. With a hypothesised ESBL prevalence of 8% [9,11,12], we needed ca 2,000 participants to reach > 80% power to detect an OR of around 1.5 (with a Type I error of 5%) [25].

### Ethical statement

This study received ethics approval from the Medical Research Ethics Committee of the University Medical Center, Utrecht (WAG/om/14/012490).

## Results

### Participants

**Figure 1:**
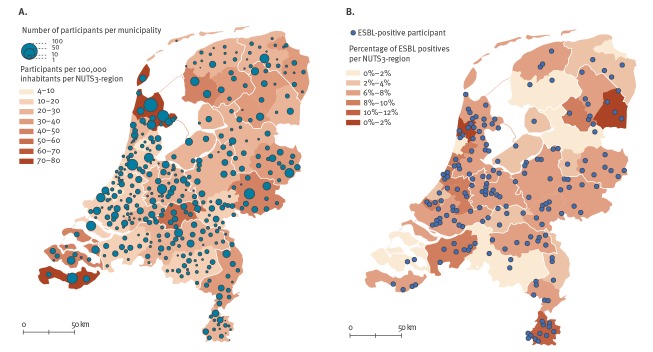
Geographical distributions of (A) the study population (n = 4,177) and (B) study participants who tested positive for carriage of extended-spectrum beta-lactamase producing Enterobacteriaceae (n = 186), the Netherlands, 2014–2016

ESBL: extended-spectrum beta-lactamase producing Enterobacteriaceae; NUTS: Nomenclature des Unités Territoriales Statistiques (nomenclature of territorial units for statistics).

In total, 47,957 individuals were invited to participate in the study, of whom 8,788 (18.3%) completed the first questionnaire, and 4,177 (8.7%) also submitted a faecal sample. Participants were distributed equally over the country (Figure 1A), with a mean age of 50 years (± standard deviation (SD): 22 years; range: 0–103 years), and 45.5% (n = 1,899) being males. Compared with the Dutch general population in 2015, the study population was – on average – older (50 vs 41 years), more frequently with both parents born in the Netherlands (97.5% vs 78.3%), and living in less densely populated areas (Supplement S3). Antibiotic use in the past 6 months was reported by 450 participants (n = 3,943 respondents to this question; 11.4%), 334 (n = 4,132; 8.1%) had been hospitalised in the last year, and 650 (n = 4,099; 15.9%) participants used proton pump inhibitors (PPI) in the past 6 months. There were 1,787 (n = 4,130; 43.3%) participants who lived in a household with children (< 18 years old), and 733 (n = 3,333; 22.0%) participants had or were children in the household who went to day care. Descriptive statistics of all 79 putative risk factors are presented in Supplement S4; Supplement S5 presents all variables that were analysed stratified by ESBL-E status, and Table 1 contains the variables finally used in logistic regression analyses.

Panel A: The numbers of study participants per municipality are shown in groups of 100, 50, 10 or 1. In addition, the numbers of participants per 100,000 inhabitants are presented per NUTS-3 region, which is the classification system of the European Union for the purpose of compiling regional statistics.

Panel B: The percentage of ESBL-E-positive participants is shown per NUTS-3 region. The ESBL-E-positive participants are distributed all over the country, and we did not observe significant differences per province in ESBL prevalence.

**Table 1:** Assessment of risk factors associated with ESBL-E carriage in persons recruited from the general community by uni- and multivariate logistic regression analyses and their population attributable risk, the Netherlands, 2014–2016 (n = 4,177)

Variable			ESBL- N = 3,991		ESBL- %	ESBL+ N = 186		ESBL+ %	Univariate OR (95% CI)	Multivariate OR (95% CI)^a^	PAR (95% CI)
			**n**	**Total responding**		**n**	**Total responding**				
**Sex (male)**			1,805	3,991	45.2	94	185	50.8	1.25 (0.93−1.68)	1.51 (1.03−2.21)	17.2 (1.5–27.8)
**Born outside the Netherlands**			3,865	3,991	96.8	172	185	93.0	2.32 (1.28−4.19)	1.99 (1.16−4.54)	46.3 (12.8–72.5)
**Social economic status (SES)^b^**	Low		865	3,970	21.8	38	183	20.8	Reference	NA	
	Intermediate		2,231	3,970	56.2	94	183	51.4	1.33 (0.86−2.04)		
	High		874	3,970	22.0	51	183	27.9	0.96 (0.65−1.41)		
**Age group in years**	0–4		173	3,991	4.3	7	184	3.8	Reference	NA	
	5–12		274	3,991	6.9	9	184	4.9	0.81 (0.30−2.22)		
	13–19		136	3,991	3.4	7	184	3.8	1.27 (0.44−3.71)		
	20–39		501	3,991	12.6	20	184	10.9	0.99 (0.41−2.38)		
	40–64		1,738	3,991	43.5	90	184	48.9	1.28 (0.58−2.81)		
	65–79		1,025	3,991	25.7	46	184	25.0	1.11 (0.49−2.50)		
	≥ 80		144	3,991	3.6	5	184	2.7	0.86 (0.27−2.76)		
**Season^c^**	Autumn		1,242	3,990	31.1	62	184	33.7	Reference	NA	
	Winter		987	3,990	24.7	36	184	19.6	0.89 (0.59−1.33)		
	Spring		835	3,990	20.9	45	184	24.5	0.73 (0.48−1.11)		
	Summer		926	3,990	23.2	41	184	22.3	1.08 (0.73−1.60)		
**Level of education^d^**	Low		610	3,908	15.6	27	178	15.2	Reference	NA	
	Intermediate		1,850	3,908	47.3	67	178	37.6	0.82 (0.52−1.29)		
	High		1,448	3,908	37.1	84	178	47.2	1.31 (0.84−2.04)		
**Eat in restaurants > 20 times a year**			398	3,946	10.1	33	183	18.0	1.96 (1.33−2.90)	1.70 (1.04−2.76)	7.4 (0.7–11.5)
**Children < 12 years old in the household**			966	3,938	24.5	33	182	18.1	0.68 (0.46–1.00)	NA	
**Ate pork**			1,506	1,860	81.0	71	95	74.7	0.70 (0.43−1.13)	0.62 (0.32−1.07)	NA
**Used antibiotics in the past**	Never		549	3,773	14.6	15	170	8.8	Reference	NA	
	< 6 months ago		422	3,773	11.2	28	170	16.5	2.12 (1.02−4.40)	2.05 (1.05−4.03)	8.7 (0.3–12.8)
	6–12 months ago		291	3,773	7.7	13	170	7.6	1.66 (0.96−2.87)	1.65 (0.71−3.81)	NA
	> 12 months ago		2,511	3,773	66.6	114	170	67.1	1.64 (0.77−3.48)	1.20 (0.64−2.23)	NA
**Animals in or around the household**			1,914	3,923	48.8	102	182	56.0	1.34 (0.99−1.81)	NA	
**Swimming in sea/ocean in the past 12 months**			1,353	3,596	37.6	80	165	48.5	1.56 (1.14−2.13)	1.63 (1.11−2.39)	18.7 (4.8–28.2)
**Swimming in open fresh water (lakes, rivers, etc.) in the past 4 weeks**			158	2,974	5.3	12	133	9.0	1.77 (0.96−3.27)	NA	
**Swimming in sea/ocean in the past 4 weeks**			169	2,979	5.7	15	135	11.1	2.08 (1.19−3.64)	NA	
**Not changing the kitchen towel on a daily basis**			2,973	3,920	75.8	159	182	87.4	2.20 (1.41−3.43)	2.19 (1.24−3.87)	47.5 (16.9–64.8)
**Travelling in the 4 weeks before faecal sample collection**			720	3,551	20.3	45	162	27.8	1.51 (1.06–2.15)	1.17 (0.76–1.81)	NA
**Travelling in the past 12 months**			2,677	3,937	68.0	136	182	74.7	1.39 (0.99−1.96)	NA	
**Travelling in the past 12 months^e^**		No	1,260	3,891	32.4	46	181	25.3	Reference	NA	
		To Europe	2,310	3,891	59.4	100	181	55.2	1.19 (0.83−1.69)	0.83 (0.54−1.29)	NA
		To Africa	57	3,891	1.5	8	181	4.4	3.85 (1.73−8.50)	3.03 (1.23−7.46)	2.9 (0.8–3.8)
		To Asia	162	3,891	4.2	22	181	12.2	3.73 (2.18−6.35)	2.00 (1.02−3.90)	6.1 (0.02–9.1)
		To North America	102	3,891	2.6	5	181	2.8	1.34 (0.52−3.46)	0.17 (0.02−3.62)	NA

CI: confidence interval; ESBL-E: extended-spectrum beta-lactamase producing Enterobacteriaceae; NA: not applicable (variable is not part of this analysis); OR: odds ratio; PAR: population attributable risk (always based on the final multivariate logistic regression model after imputation of missing values).

^a^ Always controlled for the following variables: age (0–4, 5–12, 13–19, 20–39, 40–64, 65–79, ≥ 80 years) and season^c^.

^b ^Social economic status (SES): all neighbourhoods were ranked according to their social status based on income, occupation and education level per postcode. High: the first quantile of the rank; intermediate: the second and third quantiles of the rank; low: the fourth quantile of the rank.

^c^ For the season, autumn was from September to November, winter from December to February, spring from March to May and summer from June to August.

^d ^Level of education: low: primary, lower vocational or lower secondary education; intermediate: intermediate vocational, intermediate/higher secondary education; higher: higher vocational, college and university education.

^e ^The travel destination of 46 individuals was unknown.

### Prevalence of extended-spectrum beta-lactamase producing Enterobacteriaceae carriage

ESBL-E carriage was detected in 186 individuals, yielding a crude ESBL-E prevalence of 4.5% (95% CI: 3.9–5.2%) and an overall prevalence (adjusted for age, sex, ethnicity, and degree of urbanisation, according to the reference population) of 5.0% (95% CI: 3.4–6.6%). Nearly all individuals carried *E. coli* (n = 174; 93.5%), with 10 persons carrying *K. pneumoniae* (5.4%) and two carrying *Enterobacter cloacae* complex isolates (1.1%). Three persons carried two different isolates that were ESBL-E positive (the total number of isolates = 189). ESBL-E carriers were distributed equally over the country (Figure 1B). CPE were not detected in any of the 1,758 samples tested.

### Risk factors

Risk factors for ESBL-E carriage are presented in Table 1. Those significantly associated with carriage in the multivariate models were: being born outside the Netherlands (OR: 1.99; 95% CI: 1.16–4.54), eating in restaurants > 20 times/year (OR: 1.70; 95% CI: 1.04–2.76), antibiotic use in the past 6 months (OR: 2.05; 95% CI: 1.05–4.403), swimming in sea/ocean in the past 12 months (OR: 1.63; 95% CI: 1.11–2.39), not changing kitchen towels daily (OR: 2.19; 95% CI: 1.24–3.87), travelling to Africa (OR: 3.03; 95% CI: 1.23–7.46) or Asia (OR: 2.00; 95% CI: 1.02–3.90) in the past 12 months and male sex (OR: 1.51; 95% CI: 1.03–2.21). The latter though, was only statistically significant after correcting for ‘eating pork’ as a covariate in the multivariate model. Comparison between complete record (n = 3,571) and imputed dataset analyses yielded the same risk factors (data not shown).

Several travel-related risk factors were linked. For instance, being ‘born outside the Netherlands’ was associated with ‘travelling in the past 12 months’ (Supplement S6), as well as in the 4 weeks before faecal sample collection (i.e. among individuals born outside the Netherlands 29.2% had travelled in the 4 weeks before sample collection vs 20.3% among those born in the country; chi-squared test, p = 0.025). ‘Eating in restaurants > 20 times/year‘ and ‘travelling in the past 12 months‘ were associated as well (14.1% who had eaten in restaurants >20 times/year had also travelled in the past 12 months vs 2.4% of participants who had not dined in restaurants 20 times/year; chi-squared test, p < 0.001)).

‘Not changing the kitchen towel on a daily basis’ was associated to other hygiene-related factors, like ‘not changing the dishcloth on a daily basis’ (95.1% of the participants who did not change the kitchen towel also did not change the dishcloth on a daily basis vs 47.1% who did this daily; chi-squared test, p < 0.001) and ‘not changing the toilet towel on a daily basis’ (86.6% vs 26.8%; chi-squared test, p < 0.001). Moreover, participants who did not change the kitchen towel on a daily basis were more likely to not always wash their hands before preparing food (87.6% vs 69.0%; chi-squared test, p < 0.001), after preparing meat and subsequently preparing vegetables (87.1% vs 73.9%; chi-squared test, p < 0.001) and after visiting the toilet (83.1% vs 72.4%; chi-squared test, p < 0.001).

### Predicting extended-spectrum beta-lactamase producing Enterobacteriaceae carriage

The predicted probabilities of ESBL carriage based on the risk factors identified in the final multivariable logistic regression model were used to predict a participant to be ESBL-E carrier. The highest score of the model would be obtained in 2% of the population and would have – in these individuals – a PPV of 0.26 (Supplement S7).

### Population attributable risk

The highest adjusted prevalence of ESBL-E carriage was in individuals who had travelled to Africa (11.8%) or Asia (8.4%) in the past 12 months and in those who were born outside the Netherlands (8.8%) (Table 2). Yet, as only few ESBL-E positive persons had travelled to these countries in the past 12 months, the PAR was low. In contrast, despite an adjusted prevalence of 4.8%, the risk group ‘not changing the kitchen towel on a daily basis’ had the largest PAR (47.5%). This implies that 47.5% of ESBL-E carriage could be prevented if this risk factor were eliminated and fully causally linked. For other modifiable risk factors PARs were lower (Table 1).

**Table 2:** Adjusted ESBL prevalence per subgroup of study participants according to each risk factor, the Netherlands, 2014–2016 (n = 4,177)

Risk factors	Prevalence	
	%	95% CI
**Sex**		
Female	3.6	2.7–4.4
Male	5.1	4.0–6.2
**Country of birth**		
Netherlands	4.1	3.4–4.8
Other	8.8	3.7–13.8
**Eating in a restaurant**		
≤ 20 times a year	3.9	3.2–4.6
> 20 times a year	6.5	4.0–8.9
**Used antibiotics in the past**		
Never	2.9	1.4–4.4
< 6 months ago	5.9	3.5–8.3
6–12 months ago	4.8	2.2–7.5
> 12 months ago	4.2	3.4–5.1
**Swimming in the sea/ocean in the past 12 months**		
No	3.6	2.7–4.4
Yes	5.2	4.0–6.5
**Changing the kitchen towel**		
Daily	2.3	1.2–3.4
Not daily	4.8	4.0–5.6
**Travelled (in the past 12 months)**		
No	4.5	3.0–6.0
To Europe	3.7	2.9–4.5
To Africa	11.8	3.6–20.0
To Asia	8.4	4.4–12.5
To North America	1.6	0.0–3.7

ESBL: extended-spectrum beta-lactamase.

The adjusted prevalences based on the multivariate logistic regression model are presented in this table.

### Molecular characteristics

**Figure 2:**
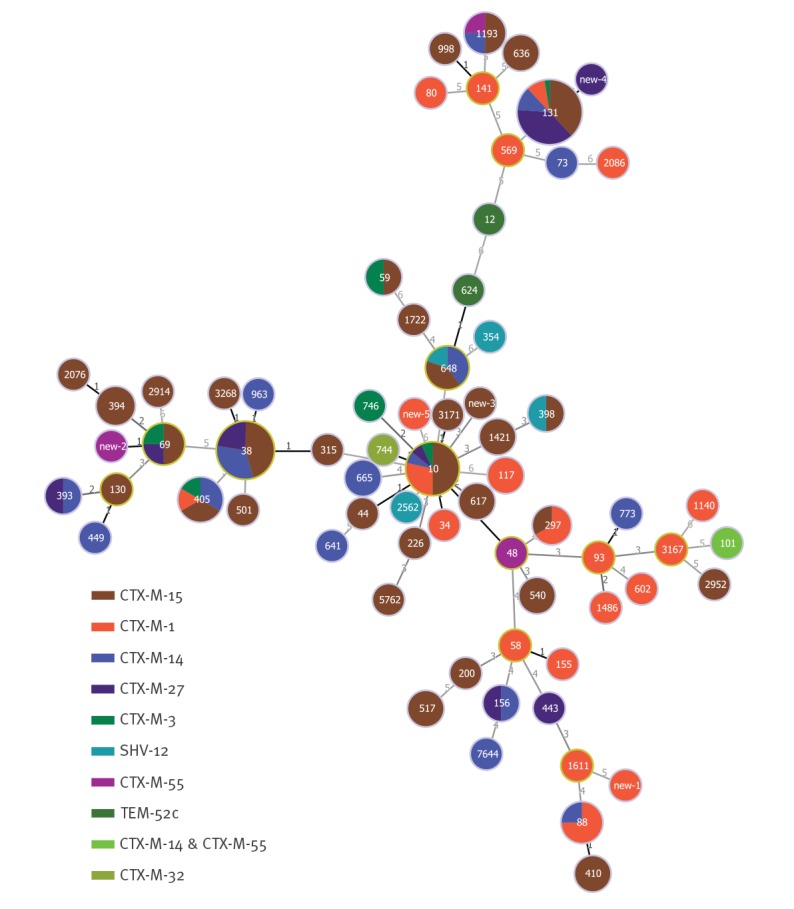
The distribution of ESBL-types among sequence types, the Netherlands, 2014–2016 (n = 189)

ESBL: extended-spectrum beta-lactamase; STs: sequence types.

Nearly half of the 189 ESBL-E isolates carried *bla*_CTX-M-15_ (n = 84; 44.4%) including 73 *E. coli* isolates, nine of the 10 *K. pneumoniae* isolates and both *Enterobacter cloacae* complex isolates. Plasmid replicons were detected in 178 isolates, yielding 29 different replicons, with IncFIB (n = 132), IncFII (n = 101), IncFIA (n = 53), and IncI1 (n = 45) being most prevalent (Supplement S8). IncI1 and FIA/FIB type plasmids were most common (n = 28 and n = 44, respectively; Supplement S9). Co-resistance among ESBL-E was common and one isolate carried *mcr-1* (Supplement S10). Prevalence of virulence factors did not differ significantly between different ESBL-producing *E. coli* genotypes (Supplement S11). The 178 *E. coli* isolates belonged to 70 different STs, of which ST131 (n = 42; 23.6%), ST38 (n = 22; 12.4%) and ST10 (n = 14; 7.9%) were most prevalent. Forty-eight STs were represented by only one isolate each. The distribution of the ESBL types among the STs is shown in Figure 2. 

Minimal spanning tree showing the distribution of the ESBL types for the STs. The size of the nodes is proportional to the number of isolates; the numbers indicate the ST. The numbers on the lines indicate the number of loci that differ between STs.

**Figure 3:**
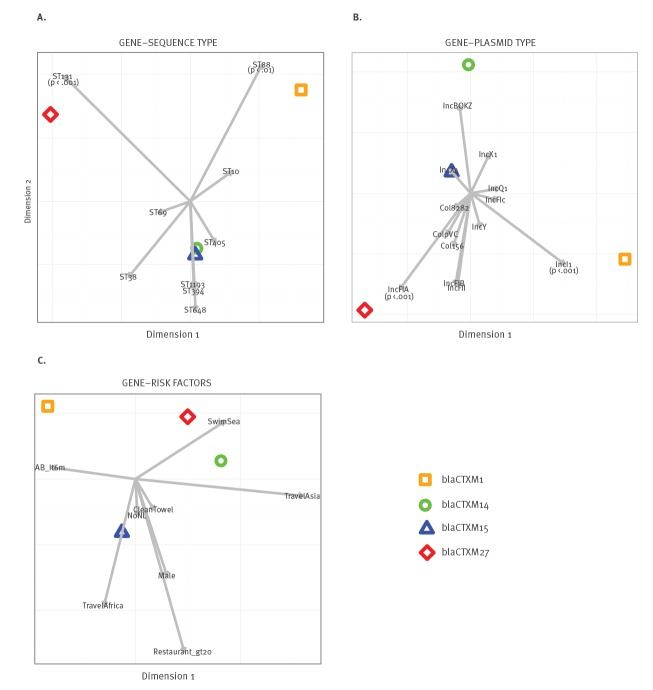
Canonical correspondence analyses (CCA) plot of ESBL genes vs (A) STs, (B) plasmid types and (C) significant risk factors, the Netherlands, 2014–2016

Canonical correspondence analysis (CCA) yielded statistically significant associations between the IncFIA plasmid and ST131 and *bla*_CTX-M-27_, and between ST88 and IncI1 and *bla*_CTX-M-1_. No significant associations were observed for any of the risk factors with genotypes, ESBL-genes or plasmids (Figure 3).

AB_lt6m: used antibiotics < 6 months ago; cleanTowel: not changing the kitchen towel on a daily basis; ESBL: extended-spectrum beta-lactamase; nonNL: born outside the Netherlands; Restaurant_gt20: eat in restaurants > 20 times a year; STs: sequence types; swimSea: swimming in sea/ocean in the past 12 months; travelAfrica: travelled to Africa in the past 12 months; travelAs: travelled to Asia in the past 12 months.

The proportion explained for the first dimension was 61%, the second 27% and the third 12% for panel A; 69%, 25% and 6%, respectively, for panel B and 50%, 35% and 15% for panel C. These canonical dimensions represent orthogonal, linear combinations of the original variables within each of the two sets (i.e. genotypes and STs) that best explain the variability both within and between these two sets of variables. The first two canonical dimensions are plotted and STs, plasmid types and risk factors significantly associated with ESBL carriage are represented as arrows directed to where their correlations with the ESBL genes specificity are maximal. The ESBL genes, STs and plasmids were plotted if n = > 10, > 2 and > 5, respectively. Significant associations (chi-squared or Fisher’s exact tests) are presented in the Figure.

## Discussion

In this cross-sectional study of a representative sample of the Netherlands’ general population, the adjusted prevalence of intestinal carriage with ESBL-producing Enterobacteriaceae was 5%. Identified risk factors were mostly travelling to Africa and Asia as well as kitchen hygiene. Among ESBL-producing *E. coli* ST131 was prevalent, associated with the IncFIA plasmid and *bla*_CTX-M-27_.

The study was set out to obtain a representative sample of the country’s general population. In all, 47,957 individuals were invited to participate and the response rate was 18.3%, which is comparable to similar studies in the Netherlands and Sweden [10,26]. The use of population registries for participant recruitment, allowed oversampling of less responsive age groups to optimise representativeness of the study population. The remaining differences between the study population and the Dutch population in age, sex, ethnicity and degree of urbanisation (Supplement S3) were used to calculate the weighted ESBL-E prevalence for the country, which was 5.0%.

The ESBL-E carriage prevalence of 5% resembles that of 4.7% among 1,033 persons in eight municipalities with the lowest and highest number of broiler farms per km^2^ [27], of 4.1% among the general population in a livestock-dense area [28] and of 3.6% in families with young children [10], all in the Netherlands. Yet, these prevalence figures are considerably lower than the 8.6% prevalence among persons registered with five family physician practices in Amsterdam [11]. Although there were small differences in culture methodology between that and the current study, these were unlikely to result in such a difference. Therefore, participant characteristics appear a more reasonable explanation. For instance, the Amsterdam-based study population travelled more often in the past 12 months (83.4%; 95% CI: 81.5–85.3%) as compared with the nationwide population in the current study (68.3%; 95% CI: 66.8–69.7%). In another Dutch study, patients were screened at hospital admission, which also yielded a higher prevalence; 8.2% (111/1,351) [29].

There are few data available from other countries for comparison. In a Swedish study that included faecal samples from 2,134 individuals in 2012–2013, the prevalence of ESBL-producing *E. coli* was 4.4% (95% CI: 3.5–5.3) [26], which is comparable to the observed prevalence in our study. In another study on the burden of infections caused by antibiotic-resistant bacteria in countries of the European Union and European Economic Area in 2015 there were large differences between countries in terms of attributable deaths and disability-adjusted life-years (DALYs) [30]. The Netherlands was among the three countries with the lowest number of attributable deaths and DALY loss, which may reflect a low prevalence of carriage with antibiotic-resistant bacteria in the general community. The Netherlands also had the lowest antimicrobial community usage with a defined daily doses (DDD) of 10.44 per 1,000 inhabitants per day (DID) in 2016 (and 10.06 in 2017 [31]), where Greece had the highest 36.3 DDD per DID [32]. Naturally, there are large differences between countries regarding antibiotic consumption, DALYS due to antibiotic resistance and probably also the prevalence of carriage in the general community.

We identified eight risk factors. Yet, all associations had low ORs. As a result, the population attributable risks of these risk factors were low. For instance, the population attributable risk of the risk factor with the highest ORs ‘travel to Africa in the past 12 months’ (OR: 3.03) was 2.9%. Several risk factors appeared to be linked. Born outside the Netherlands, eating in a restaurant > 20 times/year, swimming in sea/ocean in the past 12 months were all linked to travelling in the past 12 months. The association between travelling to Asia in the past 12 months and ESBL-E carriage confirms previous findings [33-35]. In a longitudinal analysis of ESBL-E carriage among Dutch travellers returning from hyperendemic countries, the prevalence immediately after travel was 34.3%, which had declined to 11.3% after 12 months [33]. The latter prevalence comes close to the observed 11.8% and 8.4% carriage among people who reported travelling to Africa or Asia, respectively, in the last 12 months in the current study.

Not changing the kitchen towel on a daily basis was related to other hygiene related behaviour, such as not always cleaning hands before preparing food or after visiting the toilet. These findings may result from different transmission pathways, such as direct person-to-person transmission or indirect transmission through contaminated kitchen towels (e.g. contaminated by an ESBL-E positive household member or from food contaminated with ESBL-E), which was also suggested to occur in day-care centres [36]. Furthermore, antibiotic use in the past 6 months was a risk factor for ESBL-E carriage, which also confirms previous findings [9,11].

Day-care attendance and PPI use were not risk factors in the overall analysis, but both were in previous Dutch studies [10,11,13,37]. To allow for a fair comparison, we ran additional subgroup analyses to examine the role of day-care attendance in participants living in households with children < 12 years old and the role of PPI use in the population with the highest PPI use, i.e. those > 40 years old. In the first subgroup analysis day-care attendance of children was associated ESBL-E carriage (OR: 2.17; 95% CI: 1.05–4.36), which is in line with our previous work [10]. In the population > 40 years old, ESBL-E carriage prevalence was 4.4% (105/2,395) in non-PPI and 5.3% (32/599) in PPI users (p = 0.371). Methodological differences with those studies reporting significant associations between PPI and ESBL-E carriage include collection of medication usage via GP registries [11,13], compared to self-reported medication use in the current study, and faecal sample collection at hospital admission [37], yielding a study population with more comorbidities and medication use than the general population.

Based on the risk factors identified, it was not possible to derive a prediction model that could be used to guide a screening strategy. Indeed, the highest achievable PPV of combined risk factors was 26%, which would be present in 2% of the population, implying that 90% of ESBL-E carriers would be missed. Thus, our findings cannot be used for an evidence-based screening tool for ESBL-E carriage in the community-dwelling population.

The 178 ESBL-producing *E. coli* in our study belonged to 76 different STs, with ST131 (n = 42;23.6%), ST38 (n = 22;12.4%) and ST10 (n = 14;7.9%) being the most prevalent types. Based on the dominance of ST131 in clinical ESBL-producing *E. coli* isolates, ST131 has been portrayed as a globally dominant and hypervirulent clonal lineage, characterised by co-resistance to ciprofloxacin [38]. Yet, ST131 appears to be equally dominant among ESBL-producing *E. coli* carriage isolates from community-dwelling persons, without identifiable risk factors. This finding indicates that ESBL-producing *E. coli* ST131 is a successful coloniser of the human gut, even in the absence of selective antibiotic pressure. This underscores findings from the United Kingdom, providing evidence that the frequency of *E. coli* lineages in invasive disease – in time – was driven by negative frequency-dependent selection of genomic islands [39] occurring in the absence of antibiotic selective pressure [40]. As community-acquired *E. coli* infections mostly result from spillover from intestinal carriage, dominance of ST131 in clinical isolates reflects dominance of ST131 among healthy carriers, rather than enhanced virulence.

In conclusion, the prevalence of ESBL-E among the community-dwelling population in the Netherlands is 5.0%, with a few significant risk factors related to travel and hygiene, albeit with low effect sizes. This precludes, to some extent, the identification of novel risk groups as targets for interventions. However, the low prevalence in the Netherlands does not necessitate additional measures besides those already in place in healthcare facilities. The molecular epidemiology of ESBL-E is very heterogeneous, with only a limited number of ESBL types dominating in Enterobacteriaceae in the open population, and nearly half of the *E. coli* isolates belonging to ST131.

## Supplementary Data

2403000 Supplement S1-S7

## Supplementary Data

23000 Supplement S8-S11

## References

[1] Hordijk J Schoormans A Kwakernaak M Duim B Broens E Dierikx C High prevalence of fecal carriage of extended spectrum β-lactamase/AmpC-producing Enterobacteriaceae in cats and dogs. Front Microbiol. 2013;4:242. 10.3389/fmicb.2013.00242 23966992 PMC3745002

[2] Blaak H de Kruijf P Hamidjaja RA van Hoek AHAM de Roda Husman AM Schets FM Hamidjaja R a, van Hoek AH a M, de Roda Husman AM, Schets FM. Prevalence and characteristics of ESBL-producing E. coli in Dutch recreational waters influenced by wastewater treatment plants. Vet Microbiol. 2014;171(3-4):448-59. 10.1016/j.vetmic.2014.03.007 24690376

[3] Blaak H van Hoek AH Veenman C Docters van Leeuwen AE Lynch G van Overbeek WM Extended spectrum ß-lactamase- and constitutively AmpC-producing Enterobacteriaceae on fresh produce and in the agricultural environment. Int J Food Microbiol. 2014;168-169:8-16. 10.1016/j.ijfoodmicro.2013.10.006 24211774

[4] Overdevest I Willemsen I Rijnsburger M Eustace A Xu L Hawkey P Extended-spectrum β-lactamase genes of Escherichia coli in chicken meat and humans, The Netherlands. Emerg Infect Dis. 2011;17(7):1216-22. 10.3201/eid1707.110209 21762575 PMC3381403

[5] ISISweb. ISIS-AR data. Bilthoven: RIVM. [Accessed 22 Jan 2018]. Dutch. Available from: https://www.isis-web.nl/interactieve_rapporten/bezoekvraag/

[6] Dohmen W Bonten MJM Bos MEH van Marm S Scharringa J Wagenaar JA Carriage of extended-spectrum β-lactamases in pig farmers is associated with occurrence in pigs. Clin Microbiol Infect. 2015;21(10):917-23. 10.1016/j.cmi.2015.05.032 26033669

[7] Paltansing S Vlot JA Kraakman MEM Mesman R Bruijning ML Bernards AT Extended-spectrum β-lactamase-producing enterobacteriaceae among travelers from the Netherlands. Emerg Infect Dis. 2013;19(8):1206-13. 10.3201/eid1908.130257 23885972 PMC3739527

[8] von Wintersdorff CJH Penders J Stobberingh EE Oude Lashof AM Hoebe CJ Savelkoul PH High rates of antimicrobial drug resistance gene acquisition after international travel, The Netherlands. Emerg Infect Dis. 2014;20(4):649-57. 10.3201/eid2004.131718 24655888 PMC3966371

[9] Reuland EA Overdevest IT Al Naiemi N Kalpoe JS Rijnsburger MC Raadsen SA High prevalence of ESBL-producing Enterobacteriaceae carriage in Dutch community patients with gastrointestinal complaints. Clin Microbiol Infect. 2013;19(6):542-9. 10.1111/j.1469-0691.2012.03947.x 22757622

[10] van den Bunt G Liakopoulos A Mevius DJ Geurts Y Fluit AC Bonten MJ ESBL/AmpC-producing Enterobacteriaceae in households with children of preschool age: prevalence, risk factors and co-carriage. J Antimicrob Chemother. 2017;72(2):589-95. 10.1093/jac/dkw443 27789683

[11] Reuland EA Al Naiemi N Kaiser AM Heck M Kluytmans JA Savelkoul PH Prevalence and risk factors for carriage of ESBL-producing Enterobacteriaceae in Amsterdam. J Antimicrob Chemother. 2016;71(4):1076-82. 10.1093/jac/dkv441 26755493 PMC4790620

[12] Huijbers PMC de Kraker M Graat EA van Hoek AH van Santen MG de Jong MC Prevalence of extended-spectrum β-lactamase-producing Enterobacteriaceae in humans living in municipalities with high and low broiler density. Clin Microbiol Infect. 2013;19(6):E256-9. 10.1111/1469-0691.12150 23397953

[13] Wielders CCH van Hoek AHAM Hengeveld PD Veenman C Dierikx CM Zomer TP Extended-spectrum β-lactamase- and pAmpC-producing Enterobacteriaceae among the general population in a livestock-dense area. Clin Microbiol Infect. 2017;23(2):120.e1-8. 10.1016/j.cmi.2016.10.013 27773759

[14] Mughini Gras L Smid JH Wagenaar JA de Boer AG Havelaar AH Friesema IH Risk factors for campylobacteriosis of chicken, ruminant, and environmental origin: a combined case-control and source attribution analysis. PLoS One. 2012;7(8):e42599. 10.1371/journal.pone.0042599 22880049 PMC3411806

[15] Doorduyn Y Van Pelt W Havelaar AH The burden of infectious intestinal disease (IID) in the community: a survey of self-reported IID in The Netherlands. Epidemiol Infect. 2012;140(7):1185-92. 10.1017/S0950268811001099 21943704

[16] Carattoli A Bertini A Villa L Falbo V Hopkins KL Threlfall EJ Identification of plasmids by PCR-based replicon typing. J Microbiol Methods. 2005;63(3):219-28. 10.1016/j.mimet.2005.03.018 15935499

[17] Zankari E Hasman H Cosentino S Vestergaard M Rasmussen S Lund O Identification of acquired antimicrobial resistance genes. J Antimicrob Chemother. 2012;67(11):2640-4. 10.1093/jac/dks261 22782487 PMC3468078

[18] Joensen KG Scheutz F Lund O Hasman H Kaas RS Nielsen EM Real-time whole-genome sequencing for routine typing, surveillance, and outbreak detection of verotoxigenic Escherichia coli. J Clin Microbiol. 2014;52(5):1501-10. 10.1128/JCM.03617-13 24574290 PMC3993690

[19] Carattoli A Zankari E García-Fernández A Voldby Larsen M Lund O Villa L In silico detection and typing of plasmids using PlasmidFinder and plasmid multilocus sequence typing. Antimicrob Agents Chemother. 2014;58(7):3895-903. 10.1128/AAC.02412-14 24777092 PMC4068535

[20] Larsen MV Cosentino S Rasmussen S Friis C Hasman H Marvig RL Multilocus sequence typing of total-genome-sequenced bacteria. J Clin Microbiol. 2012;50(4):1355-61. 10.1128/JCM.06094-11 22238442 PMC3318499

[21] Korndewal MJ Mollema L Tcherniaeva I van der Klis F Kroes AC Oudesluys-Murphy AM Cytomegalovirus infection in the Netherlands: seroprevalence, risk factors, and implications. J Clin Virol. 2015;63:53-8. 10.1016/j.jcv.2014.11.033 25600606

[22] Benjamini Y Hochberg Y Controlling the false discovery rate: a practical and powerful approach to multiple testing. J R Stat Soc. 1995;57:289-300.

[23] van Buuren S Groothuis-oudshoorn K mice: multivariate imputation by chained equations in R. J Stat Softw. 2011;45(3):1-67. 10.18637/jss.v045.i03

[24] Rockhill B Newman B Weinberg C Use and misuse of population attributable fractions. Am J Public Health. 1998;88(1):15-9. 10.2105/AJPH.88.1.15 9584027 PMC1508384

[25] Dean AG, Sullivan KM, Soe MM. Open Source Epidemiologic Statistics for Public Health. Atlanta: Emory University; 2013. Available from: https://www.openepi.com/SampleSize/SSCC.htm

[26] Ny S Löfmark S Börjesson S Englund S Ringman M Bergström J Community carriage of ESBL-producing Escherichia coli is associated with strains of low pathogenicity: a Swedish nationwide study. J Antimicrob Chemother. 2017;72(2):582-8. 10.1093/jac/dkw419 27798205

[27] van Hoek AHAM Schouls L van Santen MG Florijn A de Greeff SC van Duijkeren E Molecular characteristics of extended-spectrum cephalosporin-resistant Enterobacteriaceae from humans in the community. PLoS One. 2015;10(6):e0129085. 10.1371/journal.pone.0129085 26029910 PMC4451282

[28] Wielders CCH van Hoek AHAM Hengeveld PD Veenman C Dierikx CM Zomer TP Extended-spectrum β-lactamase- and pAmpC-producing Enterobacteriaceae among the general population in a livestock-dense area. Clin Microbiol Infect. 2017;23(2):120.e1-8. 10.1016/j.cmi.2016.10.013 27773759

[29] Platteel TN Leverstein-van Hall MA Cohen Stuart JW Thijsen SF Mascini EM van Hees BC Predicting carriage with extended-spectrum beta-lactamase-producing bacteria at hospital admission: a cross-sectional study. Clin Microbiol Infect. 2015;21(2):141-6. 10.1016/j.cmi.2014.09.014 25658554

[30] Cassini A Högberg LD Plachouras D Quattrocchi A Hoxha A Simonsen GS Burden of AMR Collaborative Group Attributable deaths and disability-adjusted life-years caused by infections with antibiotic-resistant bacteria in the EU and the European Economic Area in 2015: a population-level modelling analysis. Lancet Infect Dis. 2019;19(1):56-66. 10.1016/S1473-3099(18)30605-4 30409683 PMC6300481

[31] Nethmap/Maran. Nethmap/Maran 2018. 2018.

[32] European Centre for Disease Prevention and Control (ECDC). Summary of the latest data on antibiotic consumption in the European Union. Stockholm: ECDC; 2017. Available from: https://ecdc.europa.eu/en/publications-data/summary-latest-data-antibiotic-consumption-eu-2017

[33] Arcilla MS van Hattem JM Haverkate MR Bootsma MCJ van Genderen PJJ Goorhuis A Import and spread of extended-spectrum β-lactamase-producing Enterobacteriaceae by international travellers (COMBAT study): a prospective, multicentre cohort study. Lancet Infect Dis. 2017;17(1):78-85. 10.1016/S1473-3099(16)30319-X 27751772

[34] Reuland EA Sonder GJB Stolte I Al Naiemi N Koek A Linde GB Travel to Asia and traveller’s diarrhoea with antibiotic treatment are independent risk factors for acquiring ciprofloxacin-resistant and extended spectrum β-lactamase-producing Enterobacteriaceae-a prospective cohort study. Clin Microbiol Infect. 2016;22(8):731.e1-7. 10.1016/j.cmi.2016.05.003 27223840

[35] von Wintersdorff CJH Penders J Stobberingh EE Oude Lashof AM Hoebe CJ Savelkoul PH High rates of antimicrobial drug resistance gene acquisition after international travel, The Netherlands. Emerg Infect Dis. 2014;20(4):649-57. 10.3201/eid2004.131718 24655888 PMC3966371

[36] Koningstein M Leenen MA Mughini-Gras L Scholts RM van Huisstede-Vlaanderen KW Enserink R Prevalence and Risk Factors for Colonization With Extended-Spectrum Cephalosporin-Resistant Escherichia coli in Children Attending Daycare Centers: A Cohort Study in the Netherlands. J Pediatric Infect Dis Soc. 2015;4(4):e93-9. 26407274 10.1093/jpids/piv042

[37] Huizinga P Kluytmans-van den Bergh-Kl M van Rijen M Willemsen I van ’t Veer N Kluytmans J Proton Pump Inhibitor use is associated with Extended- Spectrum β-Lactamase–producing Enterobacteriaceae rectal carriage at hospital admission: a cross-sectional study. Clin Infect Dis. 2016;702:5-7. 27965302 10.1093/cid/ciw743

[38] Johnson JR Johnston B Clabots C Kuskowski MA Castanheira M Escherichia coli sequence type ST131 as the major cause of serious multidrug-resistant E. coli infections in the United States. Clin Infect Dis. 2010;51(3):286-94. 10.1086/653932 20572763

[39] McNally A Kallonen T Connor C Signatures of negative frequency dependent selection in colonisation factors and the evolution of a multi-drug resistant lineage of. bioRxiv. 2018.

[40] Kallonen T Brodrick HJ Harris SR Corander J Brown NM Martin V Systematic longitudinal survey of invasive *Escherichia coli* in England demonstrates a stable population structure only transiently disturbed by the emergence of ST131. Genome Res. 2017;27(8):1437-49. 10.1101/gr.216606.116 28720578 PMC5538559

