# Supplementary material for: Prevalence, risk factors and genetic characterisation of extended-spectrum beta-lactamase and carbapenemase-producing Enterobacteriaceae (ESBL-E and CPE): a community-based cross-sectional study, the Netherlands, 2014 to 2016
Source: Euro Surveill. 2019 Oct 10;24(41):1800594. doi: 10.2807/1560-7917.ES.2019.24.41.1800594 (PMC6794991; doi:10.2807/1560-7917.ES.2019.24.41.1800594)
Supplement: Supplement S1-S7 [file 1800594_SupplementS1-S7.pdf]

This supplementary material is hosted by Eurosurveillance as supporting information alongside the article: "Prevalence, risk factors and genetic characterisation of extended-spectrum beta-lactamase and carbapenemase-producing Enterobacteriaceae (ESBL-E and CPE): a community-based cross-sectional study, the Netherlands, 2014 to 2016", on behalf of the authors, who remain responsible for the accuracy and appropriateness of the content. The same standards for ethics, copyright, attributions and permissions as for the article apply. Supplements are not edited by Eurosurveillance and the journal is not responsible for the maintenance of any links or email addresses provided therein.

Supplement S1.

Prevalence, risk factors and genetic characterization of Extended-Spectrum Beta-Lactamase and carbapenemase-producing Enterobacteriaceae (ESBL-E and CPE): a community-based repeated cross-sectional study in the Netherlands from 2014 to 2016

This is the first questionnaire which was distributed to subject from the Dutch general population.

Population study to antibiotic resistance

Welcome!

Participation in this study is entirely voluntary. If you participate you have the right to revoke your consent at any time without having to give a reason for it.

If you have any questions and / or problems, you can contact the researchers of the UMCU and the RIVM by telephone or (preferably) by e-mail. You will find the contact details of the researchers in the letter you received.

Your data will remain anonymous and will not be provided to third parties.

Thank you very much for your cooperation!

These are the topics that we will ask questions about. Depending on your answers, some parts take less time to complete than others.

* General questions
* Family and household
* Health
* Antibiotics use
* Health complaints
* Education and work
* Contact with animals and meat
* Food
* Hygiene
* Travel and other activities
* Laboratory research
* Pet questions

At the end of the questionnaire we ask for your permission to use your answers for our research. Depending on the age of the invited person, we also need permission from the parent (s) / caregiver (s).

GENERAL QUESTIONS

1. Is this questionnaire completed by the invited person? (with date of birth [Date of birth])

 No.
 Yes

2. What is your relationship with the invited person?

Do you want to fill in the questions so that it is correct for the invited person?

 Parent / caretaker
 Brother / sister
 Son / daughter
 Partner
 Other ___________

3. Is the date of birth of the invited person:

[Date of birth]

 This is correct
 This is not correct. It should be: ___________

4. What is your country of birth?

(If your country of birth is not in the list, choose the 'Other' option at the bottom)

5. My country of birth is:

____

FAMILY AND HOUSEHOLD

The following questions are about your household (the household of the person with [Date of birth] as date of birth)

6. How many persons (living at home) does your household have?

(including yourself / addressee)

 1
 2
 3
 4
 5
 6
 7

☐ 8
☐ 9
☐ 10
☐ More than 10

7. What is your living situation?

☐ I live in a single-family house (house, semi-detached house, detached house)
☐ I live in an apartment
☐ I live in a student residence
☐ I live in a nursing or care home
☐ Other, namely: ___________
☐ I live on a farm

8. Are there children (living at home) in your household?

☐ No.
☐ Yes

9. How many children (living at home) does your household have?

☐ 1
☐ 2
☐ 3
☐ 4
☐ 5
☐ 6
☐ 7
☐ 8
☐ More than 8

10. How old are the children in your household?

Multiple answers possible

☐ 0 to 12 months
☐ Between 1 and 2 years
☐ 3 to 12 years
☐ 13 to 18 years
☐ Older than 18 years

11. Are there children in your household who go to a day care center?

(multiple answers possible)

☐ No.
☐ Yes, the invited person
☐ Yes, a child from the household

12. Are there children in your household who go to a playgroup/kindergarten?

(multiple answers possible)

□ No.
□ Yes, the invited person
□ Yes, a child from the household

13. Are there children in your household who go to a guest parent?

(multiple answers possible)

□ No.
□ Yes, the invited person
□ Yes, a child from the household

14. How many children in your household wear diapers?

□ 0
□ 1
□ 2
□ 3
□ More than 3

15. Does someone smoke in your household?

□ No.
□ Yes, outdoors
□ Yes, indoors
□ I do not know

16. Which of the options below applies to you?

(multiple answer options possible)

□ My home and / or work address is near a forest
□ My home and / or work address is located near a city park / city forest
□ My home and / or work address is next to a pasture
□ My home and / or work address is located near a cropland
□ My home and / or work address is near water (sea, lake, river, stream, canal, etc.)
□ None of the above

HEALTH

Below are questions about your health (the person with date of birth [Date of birth])

17. Have you been admitted to a healthcare institution in the past 12 months? (1 night or more)

(Multiple answers possible)

□ No.
□ Yes, in a hospital
□ Yes, in a nursing home
□ Yes, in a care home

□ Yes, in a rehabilitation center
□ Yes, otherwise ___________

18. How many times have you been admitted in the past 12 months?

(1 night or more)

□ 1
□ 2
□ 3
□ 4
□ 5
□ 6
□ 7
□ 8
□ 9
□ 10
□ More than 10 times

19. Was / were the admission (s) abroad?

□ This was in the Netherlands
□ This was abroad

20. In which country (s) were you admitted?

(multiple answers possible)

21. What type of department were you admitted to?

(multiple answers possible)

□ On a short stay (short-stay department)
□ In a general nursing ward (eg oncology, urology, surgery, internal, nephrology, lung, rheumatism, stomach, intestine and liver, cardiology department, etc.)
□ In an intensive care unit (IC) or cardiac monitoring unit (CCU)
□ Other, namely ___________

22. Was one of the other family members (living at home) admitted in the last 12 months (1 night or more)?

(Multiple answers possible)

□ No.
□ Yes, in a hospital
□ Yes, in a nursing home
□ Yes, in a care home
□ Yes, in a rehabilitation center
□ Yes, otherwise ___________

23. Which long-term health complaints and / or illnesses apply to you (with date of birth [Date of birth])

(multiple answers possible)

 Disease of the gastrointestinal tract
 Food allergy
 Diabetes (diabetes)
 Absence of the spleen
 Liver disease
 Kidney disease
 Heart and / or vascular diseases
 Cancer
 Immune disorder
 Rheumatism
 Lung disease
 Other, namely ___________
 No complaints

24. Have you had medication on doctor's prescription in the past 6 months?

(Multiple answers possible)

 No.
 Yes, medication due to an infection (eg antibiotics)
 Yes, antacids (such as omeprazole, pantoprazole, lansoprazole, rabeprazole, esomeprazole)
 Yes, cholesterol-lowering drugs (such as simvastatin, pravastatin, atorvastatin)
 Yes, laxatives (eg colophort, endofalk, movicolon, molaxole)
 Yes, antihypertensive drugs (such as betaxolol, valsartan, amlodipine, etc.)
 Yes, ADHD medication (like ritalin)
 Yes, antidepressants (such as venlafaxine, lithium, MAO inhibitors, etc.)
 Yes, sedatives and tranquillizers (such as diazepam, oxazepam, temazepam, etc.)
 Yes, antidiabetics (such as metformin, glimepiride, levemir, novorapid, etc.)
 Yes, chemotherapy
 Yes, contraceptive pill (for women only)
 Yes, otherwise ___________

ANTIBIOTIC USAGE

Antibiotics are prescribed for treatment of certain infections. The doctor prescribes an antibiotic in a certain amount and for a certain number of days. This is called an antibiotic treatment.

Examples of antibiotics include penicillin (Penidural®), pheneticillin (Broxil®), flucloxacillin (Floxapen®), amoxicillin, amoxicillin-clavulanic acid (Augmentin®), nitrofurantoin (Furadantine®, Furabid®), doxycycline, azithromycin (Zithromax®) and ciprofloxacin

(Ciproxin®). It is possible that you have used or know antibiotics that are not listed here. There are many more antibiotics on the market.

PAY ATTENTION! The following questions are about the person invited in the letter (with date of birth [Date of birth])

25. When did you use antibiotics for the last time?

☐ Never
☐ The last time was less than a month ago
☐ The last time was 1 to 3 months ago
☐ The last time was 4 to 6 months ago
☐ The last time was 6 months to a year ago
☐ The last time was more than a year ago

26. How often have you used an antibiotic treatment in the last 12 months?

(If a treatment is extended, you can consider the total course as 1 time)

☐ 1 time
☐ 2 times
☐ 3 times
☐ 4 times
☐ 5 times
☐ More than 5 times

27. What was the name of the last used antibiotic?

☐ penicillin (Penidural®)
☐ pheneticillin (Broxil®)
☐ flucloxacillin (Floxapen®)
☐ amoxicillin
☐ amoxicillin clavulanic acid (Augmentin®)
☐ nitrofurantoin (Furadantine®, Furabid®)
☐ doxycycline
☐ azithromycin (Zithromax®)
☐ ciprofloxacin (Ciproxin®)
☐ ceftriaxone
☐ Other, namely ___________
☐ I do not know

28. What was the dose of this antibiotic (per intake) in mg?

☐ 50 mg
☐ 100 mg
☐ 200 mg
☐ 250 mg
☐ 300 mg
☐ 400 mg
☐ 500 mg
☐ 600 mg
☐ Other, namely ___________

☐ I do not know

29. How many days did you use the last prescribed antibiotic treatment?

☐ 1
☐ 2
☐ 3
☐ 4
☐ 5
☐ 6
☐ 7
☐ 8
☐ 9
☐ 10
☐ 11
☐ 12
☐ 13
☐ 14
☐ More than 14
☐ I do not know

30. How many times a day did you take your last antibiotic treatment?

☐ 1
☐ 2
☐ 3
☐ 4
☐ More than 4 times
☐ I do not know

31. For which infection have you been prescribed the last antibiotic treatment?

☐ Urinary tract infection (bladder infection)
☐ Pneumonia
☐ Laryngitis
☐ Gastrointestinal infection
☐ Other, namely ___________

32. How many times have you had a bladder infection during the past 6 months?

☐ 1
☐ 2
☐ 3
☐ 4
☐ More than 4

33. How did you obtain the last antibiotic treatment?

☐ By prescription from the GP
☐ By prescription from the attending physician at the hospital
☐ By prescription of dentist
☐ By prescription via the traveler's consultation

- By prescription of relative / family member
- Via internet without a prescription
- Abroad without a prescription
- From someone else's leftover
- Other, namely ___________

34. With the last antibiotics treatment, did someone tell you how to use the antibiotic?

(multiple answers possible)

- No.
- Yes, the doctor or assistant of the general practitioner
- Yes, the attending doctor or assistant in the hospital
- Yes, the dentist or dental assistant
- Yes, the pharmacist or pharmacist's assistant
- Yes, someone else, namely ___________

35. Did you use the last antibiotic treatment according to the package leaflet?

- No.
- Partially
- Yes
- I do not know

36. Have you completed the last antibiotic treatment?

So all tablets, pills or drinks were consumed. It may be that you have something left over (in the case of a drink for example), but that you have completed the prescribed antibiotic treatment according to prescription.

- No.
- Yes
- Treatment still ongoing

37. What was the main reason why you did not complete the course?

(multiple answers possible)

- My complaints were gone
- I suffered from side effects
- I have been prescribed another antibiotic
- I stopped on the advice of my doctor
- I stopped on the advice of my partner / friend / family member
- Other, namely ___________

38. What was the name of the second-to-last used antibiotic?

- penicillin (Penidural®)
- pheneticillin (Broxil®)
- flucloxacillin (Floxapen®)
- amoxicillin

□ amoxicillin clavulanic acid (Augmentin®)
□ nitrofurantoin (Furadantine®, Furabid®)
□ doxycycline
□ azithromycin (Zithromax®)
□ ciprofloxacin (Ciproxin®)
□ ceftriaxone
□ Other, namely ___________
□ I do not know

39. What was the dose of the second-last antibiotic (per intake) in mg?

□ 50 mg
□ 100 mg
□ 200 mg
□ 250 mg
□ 300 mg
□ 400 mg
□ 500 mg
□ 600 mg
□ Other, namely ___________
□ I do not know

40. How many days did you use the second-to-last antibiotic treatment prescribed to you?

□ 1
□ 2
□ 3
□ 4
□ 5
□ 6
□ 7
□ 8
□ 9
□ 10
□ 11
□ 12
□ 13
□ 14
□ More than 14
□ I do not know

41. How many times a day did you take your second-to-last antibiotic treatment?

□ 1
□ 2
□ 3
□ 4
□ More than 4 times
□ I do not know

42. What other antibiotics have you taken in addition to the aforementioned, in the past 12 months?

(more answers possible)

- penicillin (Penidural®)
- pheneticillin (Broxil®)
- flucloxacillin (Floxapen®)
- amoxicillin
- amoxicillin clavulanic acid (Augmentin®)
- nitrofurantoin (Furadantine®, Furabid®)
- doxycycline azithromycin (Zithromax®)
- ciprofloxacin (Ciproxin®)
- ceftriaxone
- Other, namely ___________
- I do not know

43. Have you ever preserved the remains of an antibiotic treatment?

- No.
- Yes
- I do not know

44. Do you ever use leftovers of antibiotics for the treatment of complaints in yourself, your family members or pets?

If yes, multiple answers possible

- No.
- Yes, for myself
- Yes, for one or more of my family members
- Yes, for one or more of my pets

HEALTH COMPLAINTS

Below you will be asked whether you have had health complaints in the past 4 weeks and, if so, what complaints were involved.

45. Please tick the complaints that you have suffered from in the last 4 weeks

(multiple answers possible)

- Vomiting
- Nausea
- Abdominal pain / abdominal cramps
- Mucus in the stool
- Blood in the stool
- Light (decolored) stool
- Constipation
- Diarrhea (at least 3 times a day)
- Fever (at least 38 degrees)

- Sluggish or flu-like feeling
- Cold
- Shortness of breath
- Long-term coughing
- Sore throat
- Nausea
- Headache
- Muscle pain (not through sports)
- Pain in the chest
- Prolonged sneezing (not by hay fever)
- Earache
- Running ear
- Inflamed eye
- Other, namely ___________
- None of the above complaints

46. How many days (approximately) did you suffer from these complaints in the past 4 weeks?

- 1 day
- 2 days
- 3 days
- 4 days
- 5 days
- 6 days
- 7 days
- 8 days
- 9 days
- 10 days
- 11 days
- 12 days
- 13 days
- 14 days
- More than 14 days
- I do not know

47. Did you have contact with a doctor in the past 4 weeks due to your complaints?

- No.
- Yes, once
- Yes, twice
- Yes, 3 times
- Yes, 4 times
- Yes, 5 times
- Yes, more than 5 times

48. Did you have one or more hospital admissions during the past 4 weeks as a result of your complaints?

- No.
- Yes, once
- Yes, twice

☐ Yes, 3 times
☐ Yes, 4 times
☐ Yes, 5 times
☐ Yes, more than 5 times

49. Have you taken medication in the past 4 weeks to relieve your symptoms or as a remedy?

☐ No.
☐ Yes

50. Could you only receive this medication on prescription from a doctor?

☐ No.
☐ Yes
☐ I do not know

51. What was the name of this medicine?

☐ Paracetamol
☐ Ibuprofen
☐ Otherwise ___________
☐ I do not know

52. How many days did you take this medicine?

☐ 1 day
☐ 2 days
☐ 3 days
☐ 4 days
☐ 5 days
☐ 6 days
☐ 7 days
☐ 8 days
☐ 9 days
☐ 10 days
☐ 11 days
☐ 12 days
☐ 13 days
☐ 14 days
☐ More than 14 days
☐ I do not know

53. Have you taken any other medication in the past 4 weeks to relieve your symptoms or as a remedy?

☐ No.
☐ Yes
☐ I do not know

54. Could you only receive this medication on prescription from a doctor?

 No.
 Yes
 I do not know

55. What was the name of this medicine?

 Paracetamol
 Ibuprofen
 Other, namely ___________
 I do not know

56. How many days did you take this medicine?

 1 day
 2 days
 3 days
 4 days
 5 days
 6 days
 7 days
 8 days
 9 days
 10 days
 11 days
 12 days
 13 days
 14 days
 More than 14 days
 I do not know

57. Have any samples been taken in the past 4 weeks due to your complaints?

(eg blood, urine or stool)

 No.
 Yes

58. What samples were taken?

(multiple answers possible)

 Blood
 Urine
 Feces
 Otherwise ___________

59. What was the outcome of the stool examination?

(multiple answers possible)

- Nothing found
- Campylobacter
- Salmonella
- Shigella
- Yersinia
- Vibrio
- Clostridium difficile
- Aeromonas
- Escherichia coli
- Rotavirus
- Norovirus
- Adenovirus types 40/41
- Astrovirus
- Entamoeba histolytica
- Giardia lamblia
- Cryptosporidien
- Cyclospora
- Other, namely: ___________
- I do not know

60. Have you stayed overnight abroad in the 7 days before the start of your complaints?

- No.
- Yes

61. In which country did you stay overnight in the 7 days before the start of your complaints?

62. Have you been in contact with someone with stomach and / or intestinal complaints in the past 4 weeks?

- No.
- Yes
- I do not know

EDUCATION AND WORK

The following questions are about education and work. If you fill in this questionnaire for your (foster) child under the age of 18, please fill in your own data.

63. What is your highest level of education?

- Primary school (primary education, special education)
- Lower vocational education (for example LTS, LHNO, LEAO, housekeeping school)
- Lower secondary education (eg VMBO, ULO, MULO, MAVO)
- Intermediate vocational education (eg MTS, MEAO, MHNO).
- Intermediate or higher secondary education (eg HBS, MMS, HAVO, VWO, gymnasium)
- Higher vocational education (eg HTS, HEAO, HHNO)
- University education
- No training completed

□ I would rather not answer this question

64. Do you have a paid job?

□ No.
□ Yes

65. How many hours per week do you work?

(average for flexible working hours)

approximately ___________________________

66. In which sector do you work?

(multiple answers possible)

□ Health or well-being
□ Education
□ Childcare
□ Animal care / animal welfare
□ Catering
□ Forestry / forest management
□ Agriculture
□ Livestock farming
□ Meat processing (including slaughterhouse)
□ Transport of animals / agricultural products
□ Dairy industry
□ Wool, felt or leather processing
□ Construction / road construction
□ Trade
□ Business services
□ Government and politics
□ Industry and energy
□ Waste processing
□ Water management
□ Pest and pest control
□ Otherwise

67. Do you have a profession in which you come into contact with groups of children?

(For example, teacher, crèche employee, consultation nurse, etc.)

□ No.
□ Yes

68. Do you have direct contact with the patients / clients / residents during your work in health or social care?

(eg, nurse, doctor)

□ No.
□ Yes

69. What is your profession?

□ Assisting role
□ Caring
□ Nurse (level 4 or 5)
□ Nursing specialist
□ Physical therapist
□ Doctor
□ Doctor's assistant (e)
□ Ergo therapist
□ Dietitian
□ Other, namely ___________

70. In what kind of institution do you work?

(multiple answers possible)

□ Hospital
□ Nursing home
□ Care home
□ Rehabilitation center
□ GP practice
□ Home care
□ Consultancy
□ GGD (community health service)
□ GGZ (mental health care)
□ Otherwise ___________

71. Do you have a profession in which you come into contact with farm animals or pets?

(eg cattle farmer, veterinarian, dog groomer, etc.)

□ No.
□ Yes

72. With which animals do you have occupational contact?

(multiple answers possible)

□ Dog (s)
□ Cat (s)
□ Rabbit (s)
□ Mouse / mice (as a pet)
□ Rat (s) (as a pet)
□ Guinea pig (s)
□ Hamster (s)

□ Cow/cattle
□ Sheep
□ Chicken (s)
□ Birds (as pets)
□ Pig (s)
□ Goat (s)
□ Deer
□ Horse (s) / pony (s)
□ Donkey (s)
□ Reptiles
□ Fish
□ Insects
□ Other, namely ___________

73. Do you have an occupation where you prepare food for others?

□ No.
□ Yes

74. Do you have a profession where you come into contact with raw meat?

(for example butcher, meat processing industry, etc)

□ No.
□ Yes

75. With which raw animal product (s) do you come into contact?

□ Beef
□ Mutton
□ Chicken meat
□ Pork
□ Goat meat
□ Rabbit meat
□ Horse meat
□ Other, namely ___________

CONTACT WITH ANIMALS AND MEAT

The following questions are about contact with pets, farms animals, and meat.

PAY ATTENTION! These questions are addressed to the person with date of birth [Date of birth]

76. Do you keep farm animals or pets in or around your home?

(multiple answers possible)

□ No.
□ Dog (s)
□ Cat (s)
□ Rabbit (s)

- Mouse / mice (as a pet)
- Rat (s) (as a pet)
- Guinea pig (s)
- Hamster (s)
- Cow/cattle
- Sheep
- Chicken (s)
- Other poultry (as farm animals)
- Birds (as pets)
- Pig (s)
- Goat (s)
- Deer
- Horse (s) / pony (s)
- Donkey (s)
- Reptiles
- Fish
- Insects
- Other, namely: ___________

77. Have you had direct contact with farm animals or pets in the past four weeks?

(direct contact means touching your pet, for example by petting or cuddling with your pet)

- No.
- Yes
- I do not know

78. Which farm animals or pets did you have contact with in the past 4 weeks?

(multiple answers possible)

- Dog (s)
- Cat (s)
- Rabbit (s)
- Mouse / mice (as a pet)
- Rat (s) (as a pet)
- Guinea pig (s)
- Hamster (s)
- Cow/cattle
- Sheep
- Chicken (s)
- Other poultry (as farm animals)
- Birds (as pets)
- Pig (s)
- Goat (s)
- Deer
- Horse (s) / pony (s)
- Donkey (s)
- Reptiles
- Fish
- Insects
- Other, namely: ___________

79. Have you seen rat and / or mouse excreta in your area in the past four weeks?

(multiple answers possible)

□ No.
□ Yes, indoors
□ Yes, around / near the house
□ At another location, namely ___________

80. Have you been in contact with the following animals / (animal) products in the past four weeks?

if you have had contact with several animal species, please mention all animal species

□ Manure / excrement of farm animals (eg during cleaning of pens / stalls or fertilizing your garden)
□ Compost
□ Hay / straw
□ Dead animals, namely: (animal species) ___________
□ Animals that recently gave birth (dead or alive), namely (animal species) ___________
□ Placenta material, namely: (animal species) ___________
□ Untreated wool, namely: (animal species) ___________
□ Animal fur or skin of a dead animal, namely: (animal species) ___________
□ None of the above

DIET

The following questions are about the eating habits of the person addressed with the date of birth [Date of birth]

81. Which eating habits apply to you / the addressee?

(gelatin is a binder product obtained from animal products. In non-vegetarian cheese, rennet obtained from calf stomach is processed)

□ You never eat products for which an animal has to be killed and you do not eat any other animal products (such as dairy, eggs and honey)
□ You never eat products for which an animal has to be killed, such as meat, fish, cheese and gelatin
□ You never eat meat and fish, but (sometimes) cheese and / or products that contain gelatin
□ You never eat meat, but (sometimes) fish, cheese and / or products in which gelatin is processed
□ You eat meat and / or fish, but do not eat meat and / or fish one or more days a week
□ You eat meat daily
□ None of the above

82. How often do you eat the following meat products?

[Never] [Less often than once a week] [1-3 Times a week] [4-6 Times a week] [7-8 Times a week] [More than 8 times a week]
1. Chicken ☐ ☐ ☐ ☐ ☐ ☐
2. Pork ☐ ☐ ☐ ☐ ☐ ☐
3. Beef ☐ ☐ ☐ ☐ ☐ ☐
4. (Semi) raw meat products (for example steak, minced meat, filet americain, roast beef, raw ham, beef sausage) ☐ ☐ ☐ ☐ ☐ ☐

83. How often do you eat the following fish products?

[Never] [Less often than once a week] [1-3 Times a week] [4-6 Times a week] [7-8 Times a week] [More than 8 times a week]
1. Fish ☐ ☐ ☐ ☐ ☐ ☐
2. Shellfish (such as mussels, oysters) ☐ ☐ ☐ ☐ ☐ ☐
3. Crustaceans (such as crab, lobster, shrimp) ☐ ☐ ☐ ☐ ☐ ☐

84. How often do you eat / drink the following products?

[Never] [Less often than once a week] [1-3 Times a week] [4-6 Times a week] [7-8 Times a week] [More than 8 times a week]
1. Heated eggs ☐ ☐ ☐ ☐ ☐ ☐
2. Raw eggs ☐ ☐ ☐ ☐ ☐ ☐
3. Dairy products ☐ ☐ ☐ ☐ ☐ ☐

85. How often do you eat the following products?

[Never] [Less often than once a week] [1-3 Times a week] [4-6 Times a week] [7-8 Times a week] [More than 8 times a week]
1. Vegetable (products)? ☐ ☐ ☐ ☐ ☐ ☐
2. Fruit (products)? ☐ ☐ ☐ ☐ ☐ ☐
3. Raw vegetables (eg lettuce, cucumber, tomato, carrot, garden herbs)? ☐ ☐ ☐ ☐ ☐ ☐

86. Did you eat meat products that were raw or not properly cooked in the past week?

Raw food means that it is not cooked/baked. Not well cooked meat means that it is still pink / red on the inside and / or bleeding / red juices are leaking.

(multiple answers possible)

☐ No.
☐ Yes, beef
☐ Yes, pork
☐ Yes, chicken meat
☐ Yes, turkey meat
☐ Yes, organ meat
☐ Yes, game meat
☐ Yes, otherwise ___________

87. If you eat meat, this is usually

☐ Regular meat from the butcher, supermarket or toko

□ Organic meat from the butcher, supermarket or toko
□ Meat from a store directly connected to a livestock farm
□ I do not know

88. If you eat vegetables, this is usually:

□ Regular vegetables from the greengrocer's, supermarket or toko
□ Organic vegetables from the organic shop, supermarket or toko
□ Vegetables from a store directly connected to a farm
□ Vegetables from a private vegetable garden
□ I do not know

89. How much serving spoons do you eat when you eat vegetables (products)?

□ Less than 1 serving spoon (less than 50 grams)
□ 1-2 Serving spoons (about 51-100 grams)
□ 3-4 Serving spoons (about 101-200 grams)
□ More than 4 serving spoons (more than 200 grams)
□ I do not know

90. How often do you eat dinner in a restaurant or other commercial food establishment annually?
(not including takeaway meals)

□ Never
□ 1 to 3 times
□ 4 to 12 times
□ 13 to 20 times
□ More than 20 times

91. At which supermarket is your grocery shopping most often done?

□ Albert Heijn
□ Aldi
□ C1000
□ Coop
□ Jumbo
□ Lidl
□ Plus
□ Nettorama
□ Hoogvliet
□ Troefmarkt
□ Attent
□ DeKaMarkt
□ Dirk van de Broek
□ Bas van de Heijden
□ Emté
□ Other, namely ___________
□ Not applicable

92. Which products are bought in the supermarket filled in above?

(multiple answers possible)

- ☐ Meat
- ☐ Fish
- ☐ Shellfish
- ☐ Vegetable
- ☐ Fruit
- ☐ None of the above

93. Is this supermarket located in your own place of residence?

- ☐ No, it is in ___________
- ☐ Yes

94. At which supermarket do you do your second most shopping in your household?

- ☐ Albert Heijn
- ☐ Aldi
- ☐ C1000
- ☐ Coop
- ☐ Jumbo
- ☐ Lidl
- ☐ Plus
- ☐ Nettorama
- ☐ Hoogvliet
- ☐ Troefmarkt
- ☐ Attent
- ☐ DeKaMarkt
- ☐ Dirk van de Broek
- ☐ Bas van de Heijden
- ☐ Emté
- ☐ Other, namely ___________
- ☐ None of the above

95. Which products are bought in the supermarket filled in above?

(multiple answers possible)

- ☐ Meat
- ☐ Fish
- ☐ Shellfish
- ☐ Vegetable
- ☐ Fruit
- ☐ None of the above

96. Is this supermarket located in your own place of residence?

- ☐ No, it is in: ___________
- ☐ Yes

HYGIENE

The following questions are about kitchen and cleaning habits of yourself / the addressee and the entire household.

97. How much time is there usually between buying chilled and / or deep-frozen food and storing it in the fridge and freezer?

 Less than a quarter of an hour
 A quarter to a half hour
 Half an hour to an hour
 1 hour to 2 hours
 More than 2 hours
 I do not know

98. How often is the refrigerator cleaned?

It is possible that you do this yourself or that someone else does it. Enter what is applicable below.

 Weekly
 Monthly
 Every 3 months
 Less often than every 3 months

99. Do you wash (or the person who cooks in your household) your hands:

Note: if you do not eat meat yourself, you may still be able to prepare meat for others.

[Always] [Usually] [Sometimes] [Rarely] [Never]
1. Before you start cooking food
2. After cutting or preparing raw meat, before preparing raw vegetables for example

100. What material is the most commonly used cutting board made of?

Again, you may do this yourself or someone else does it for you. Fill in what is applicable in your household.

 Wood
 Plastic
 Glass
 Pottery
 Bamboo
 Other, namely ___________

101. Is the same cutting board used for cutting raw meat and for other food during the preparation of the same meal?

(If you do not eat meat yourself, it may still be that you prepare meat for others).

 No.

 Yes, I use the same cutting board
 Yes, I turn the shelf or wash it off in between
 Not applicable, I do not use a cutting board

102. Is the knife that is used for raw meat also used for cutting other foods?

(if you do not eat meat yourself, it may still be that you prepare meat for others)

 No.
 Yes, and I clean the knife in between
 Yes, and I do not clean the knife in between
 Not applicable

103. How often is there on average:

(with clean is meant here ‘dishwasher clean’)

[Every day] [Every other day] [More than once a week] [once every 1 to 2 weeks once] [every 3 to 4 weeks] [Less than once a month] [Not applicable]
1. A clean towel for the kitchen in your household? (towel with the aim to dry hands)
2. A clean dishcloth or scourer in your household?
3. A clean toilet towel in your household?

104. Do you wash your hands after a toilet visit?

 Always
 Usually
 Sometimes
 Rarely
 Never
 Not applicable (for example, wearing a diaper)

105. How often is the toilet in your house cleaned?

 Every day
 Every other day
 More than once a week
 1 time per 1 to 2 weeks
 once every 3 to 4 weeks
 Less than once a month

ACTIVITIES

The following questions are about whether you/your family undertook activities and, if so, what activities.

106. Have you been abroad in the past 12 months?

(1 night or more)

 No.

☐ Yes

107. How often have you been abroad in the past 12 months?

☐ 1 time
☐ 2 times
☐ 3 times
☐ 4 times
☐ 5 times
☐ 6 times
☐ More often than 6 times

108. To which country were you most recently in the past 12 months (1st country)?

109. What was the purpose of this trip?

☐ Holiday trip
☐ Work-related journey
☐ Study-related journey
☐ Family / friends visit
☐ Other, namely ___________

110. How long did you stay there?

☐ Less than a week
☐ 1-2 weeks
☐ 2-3 weeks
☐ 4-5 weeks
☐ 5-8 weeks
☐ 9-10 weeks
☐ 10-12 weeks
☐ More than 12 weeks

111. In which month did you return from your trip?

☐ January
☐ February
☐ March
☐ April
☐ May
☐ June
☐ July
☐ August
☐ September
☐ October
☐ November
☐ December

112. Fill in below to which country you have been in the past 12 months (2nd country):

113. What was the purpose of this trip?

☐ Holiday trip
☐ Work-related journey
☐ Study-related journey
☐ Other, namely ___________

114. How long did you stay there?

☐ Less than a week
☐ 1-2 weeks
☐ 2-3 weeks
☐ 4-5 weeks
☐ 5-8 weeks
☐ 9-10 weeks
☐ 10-12 weeks
☐ More than 12 weeks

115. In which month did you return from your trip?

☐ January
☐ February
☐ March
☐ April
☐ May
☐ June
☐ July
☐ August
☐ September
☐ October
☐ November
☐ December

116. Which countries have you been to, but were not able to fill in above?

117. How often have you undertaken the following activities in the past 12 months?

[0 times] [1 time] [2-3 times] [4-12 times] [more than 12 times]
Stayed overnight on a camping, camping site, or in the open air (several consecutive nights count as one time) ☐ ☐ ☐ ☐ ☐
Swimming in surface water (for example lake, river, but not in a swimming pool) ☐ ☐ ☐ ☐ ☐
Swam in salt water (sea) ☐ ☐ ☐ ☐ ☐
Been in the forest ☐ ☐ ☐ ☐ ☐
Visited a petting zoo ☐ ☐ ☐ ☐ ☐
Visited a playground ☐ ☐ ☐ ☐ ☐

118. How often did you undertake the following activities in the past 4 weeks?

If you have undertaken an activity, please fill in (approximately) how often this was in the last 4 weeks. If you have not undertaken any of the following activities, you can enter 0.

Note: this is about the last 4 weeks

[0 times] [1 time] [2 to 3 times] [4 to 12 times] [more often than 12 times]
Visited a garden center □ □ □ □ □
Worked in the garden □ □ □ □ □
Been in the forest □ □ □ □ □
Been in a vacation home or recreation park □ □ □ □ □
On a camping, camping site or in the open air overnight (several consecutive nights count as one time) □ □ □ □ □
Been on a golf course □ □ □ □ □
Swam in salt water (sea) □ □ □ □ □
Swim in surface water (eg lake, river, but not in a swimming pool) □ □ □ □ □
Visited a sauna, hot tub or swimming pool □ □ □ □ □
Showed somewhere else than at home (for example at work, sports location, with family) □ □ □ □ □
Visited a car wash □ □ □ □ □
Worked with a high pressure cleaner, plant spray or garden hose □ □ □ □ □
Worked on the heating or water pipes? □ □ □ □ □
Visited a petting zoo □ □ □ □ □
Visited a playground visited □ □ □ □ □

LABORATORY RESEARCH

Many bacteria are present in stool (feces). That is why feces is suitable for laboratory testing for the presence of (resistant) bacteria and pathogens. Everyone can carry resistant bacteria.

To gain insight in the (resistant) bacteria and pathogens you carry with you, we kindly ask you to participate in stool examination. You will be asked to collect a small amount of feces (poo) of yourself (or the addressee).

When you decide to participate, you will receive a package with all the necessary items including an instruction and a shipping envelope. You do not incur any costs yourself and you can indicate whether you would like to get the results. You would really help us by filling in the questions below.

Many thanks in advance!

119. Are you willing to cooperate in feces examination (poop examination) into resistant bacteria and pathogens?

If you fill in yes, you will receive more information about the further procedure after about a month.

□ No.
□ Yes

PERMISSION STATEMENT

In order to be able to carry out the research, it is important that the following questions are answered.

120. In what age category do you / the addressee fall?

☐ 0-12
☐ 13-17
☐ 18 and older

121. I want to be informed of the examination results and reports of the overall research results (not your personal results).

☐ No.
☐ Yes

122. I agree that the address details from this statement are used for follow-up investigation.

☐ No.
☐ Yes

123. Parent / caretaker 1: do you also agree with the above points, as entered by your child?

☐ No.
☐ Yes

124. Parent / caretaker 2: do you also agree with the above points, as entered by your child?

☐ No.
☐ Yes
☐ Not applicable

125. Parent / caregiver 2: Do you also agree with the above points, as entered by your child's other parent / caregiver (if applicable)?

☐ No.
☐ Yes
☐ Not applicable

126. What is your email address?

This is particularly important:
- if you want to participate in the stool examination
- we may contact you if there are additional questions
- you want to be kept informed of the general research results

____
____
____
____

127. Repeat email address

128. Do you have any comments?

Supplement S2.

Prevalence, risk factors and genetic characterization of Extended-Spectrum Beta-Lactamase and carbapenemase-producing Enterobacteriaceae (ESBL-E and CPE): a community-based repeated cross-sectional study in the Netherlands from 2014 to 2016

This is the second questionnaire which was distributed to the participants accompanied by a stool sample collection kit.

Brief questionnaire population study – fecal sampling moment 2

Welcome!

You have indicated that you are willing to participate in the repeated measurement of the population study to antimicrobial resistance. Thank you very much for this. Below you will find a brief questionnaire, with questions covering the period between the last questionnaire and the current faecal sampling moment (about 4 weeks). The accomplishment of the questionnaire will take you about 5 minutes. Thank you for your cooperation, it is much appreciated.
GENERAL QUESTIONS

1. Is this questionnaire completed by the invited person? (with date of birth [Date of birth])

 No.
 Yes

2. What is your relationship with the invited person?

Do you want to fill in the questions so that it is correct for the invited person?

 Parent / caretaker
 Brother / sister
 Son / daughter
 Partner
 Other ___________

3. Is the date of birth of the invited person:

[Date of birth]

 This is correct
 This is not correct. It should be: ___________

"You" refers to the invited person.

QUESTIONS ABOUT THE TIME BETWEEN THE FIRST QUESTIONNAIRE AND THE CURRENT QUESTIONNAIRE
4. Have you been admitted to a healthcare institution in the past 4 weeks? (1 night or more)

(Multiple answers possible)

 No.
 Yes, in a hospital
 Yes, in a nursing home
 Yes, in a care home
 Yes, in a rehabilitation center
 Yes, otherwise ___________

5. Was / were the admission (s) abroad?

□ This was in the Netherlands
□ This was abroad

6. What type of department were you admitted to?

(multiple answers possible)

□ On a short stay (short-stay department)
□ In a general nursing ward (eg oncology, urology, surgery, internal, nephrology, lung, rheumatism, stomach, intestine and liver, cardiology department, etc.)
□ In an intensive care unit (IC) or cardiac monitoring unit (CCU)
□ Other, namely ___________

7. Was one of the other family members (living at home) admitted to hospital in the last 4 weeks (1 night or more)?
(Multiple answers possible)

□ No.
□ Yes, in a hospital
□ Yes, in a nursing home
□ Yes, in a care home
□ Yes, in a rehabilitation center
□ Yes, otherwise ___________

8. Did you use antibiotics in the past 8 weeks or are you currently using antibiotics?
□ No.
□ Yes, this was between 4 and 8 weeks ago
□ Yes, this was less than 4 weeks ago
□ Yes, I ‘am currently using antibiotics

9. What was the name of the last used antibiotic?

□ penicillin (Penidural®)
□ pheneticillin (Broxil®)
□ flucloxacillin (Floxapen®)
□ amoxicillin
□ amoxicillin clavulanic acid (Augmentin®)
□ nitrofurantoin (Furadantine®, Furabid®)
□ doxycycline
□ azithromycin (Zithromax®)
□ ciprofloxacin (Ciproxin®)
□ ceftriaxone
□ Other, namely ___________
□ I do not know

10. What was the dose of this antibiotic (per intake) in mg?

□ 50 mg
□ 100 mg

□ 200 mg
□ 250 mg
□ 300 mg
□ 400 mg
□ 500 mg
□ 600 mg
□ Other, namely ___________
□ I do not know

11. How many days did you use the last prescribed antibiotic treatment?

□ 1
□ 2
□ 3
□ 4
□ 5
□ 6
□ 7
□ 8
□ 9
□ 10
□ 11
□ 12
□ 13
□ 14
□ More than 14
□ I do not know

12. How many times a day did you take your last antibiotic treatment?

□ 1
□ 2
□ 3
□ 4
□ More than 4 times
□ I do not know

13. For which infection have you been prescribed the last antibiotic treatment?

□ Urinary tract infection (bladder infection)
□ Pneumonia
□ Laryngitis
□ Gastrointestinal infection
□ Other, namely ___________

14. Please tick the complaints that you have suffered from in the last 4 weeks

(multiple answers possible)

□ Vomiting
□ Nausea
□ Abdominal pain / abdominal cramps

☐ Mucus in the stool
☐ Blood in the stool
☐ Light (decolored) stool
☐ Constipation
☐ Diarrhea (at least 3 times a day)
☐ Fever (at least 38 degrees)
☐ Sluggish or flu-like feeling
☐ Cold
☐ Shortness of breath
☐ Long-term coughing
☐ Sore throat
☐ Nausea
☐ Headache
☐ Muscle pain (not through sports)
☐ Pain in the chest
☐ Prolonged sneezing (not by hay fever)
☐ Earache
☐ Running ear
☐ Inflamed eye
☐ Other, namely ___________
☐ None of the above complaints

15. How many days (approximately) did you suffer from these complaints in the past 4 weeks?

☐ 1 day
☐ 2 days
☐ 3 days
☐ 4 days
☐ 5 days
☐ 6 days
☐ 7 days
☐ 8 days
☐ 9 days
☐ 10 days
☐ 11 days
☐ 12 days
☐ 13 days
☐ 14 days
☐ More than 14 days
☐ I do not know

16. Have you had direct contact with farm animals or pets in the past four weeks?

(direct contact means touching your pet, for example by petting or cuddling with your pet)

☐ No.
☐ Yes
☐ I do not know

17. Which farm animals or pets did you have contact with in the past 4 weeks?

(multiple answers possible)

□ Dog (s)
□ Cat (s)
□ Rabbit (s)
□ Mouse / mice (as a pet)
□ Rat (s) (as a pet)
□ Guinea pig (s)
□ Hamster (s)
□ Cow/cattle
□ Sheep
□ Chicken (s)
□ Other poultry (as farm animals)
□ Birds (as pets)
□ Pig (s)
□ Goat (s)
□ Deer
□ Horse (s) / pony (s)
□ Donkey (s)
□ Reptiles
□ Fish
□ Insects
□ Other, namely: ___________

18. Have you been in contact with the following animals / (animal) products in the past four weeks?

if you have had contact with several animal species, please mention all animal species

□ Manure / excrement of farm animals (eg during cleaning of pens / stalls or fertilizing your garden)
□ Compost
□ Hay / straw
□ Dead animals, namely: (animal species) ___________
□ Animals that recently gave birth (dead or alive), namely (animal species) ___________
□ Placenta material, namely: (animal species) ___________
□ Untreated wool, namely: (animal species) ___________
□ Animal fur or skin of a dead animal, namely: (animal species) ___________
□ None of the above

19. Did you eat meat products that were raw or not properly cooked in the past week?

Raw food means that it is not cooked/baked. Not well cooked meat means that it is still pink / red on the inside and / or bleeding / red juices are leaking.

(multiple answers possible)

□ No.
□ Yes, beef
□ Yes, pork

☐ Yes, chicken meat
☐ Yes, turkey meat
☐ Yes, organ meat
☐ Yes, game meat
☐ Yes, otherwise ___________

20. Have you been abroad in the past 4 weeks?

(1 night or more)

☐ No.
☐ Yes

21. What was the country of visit in the past 4 weeks?

22. What was the purpose of this trip?

☐ Holiday trip
☐ Work-related journey
☐ Study-related journey
☐ Family / friends visit
☐ Other, namely ___________

23. How long did you stay there?

☐ Less than a week
☐ 1-2 weeks
☐ 2-3 weeks
☐ 4-5 weeks
☐ 5-8 weeks
☐ 9-10 weeks
☐ 10-12 weeks
☐ More than 12 weeks

24. How often have you undertaken the following activities in the past 4 weeks?

If you have undertaken an activity, please fill in (approximately) how often this was in the last 4 weeks. If you have not undertaken any of the following activities, you can enter 0.

[0 times] [1 time] [2 to 3 times] [4 to 12 times] [more often than 12 times]
Visited a garden center ☐ ☐ ☐ ☐ ☐
Worked in the garden ☐ ☐ ☐ ☐ ☐
Been in the forest ☐ ☐ ☐ ☐ ☐
Been in a vacation home or recreation park ☐ ☐ ☐ ☐ ☐
On a camping, camping site or in the open air overnight (several consecutive nights count as one time) ☐ ☐ ☐ ☐ ☐
Been on a golf course ☐ ☐ ☐ ☐ ☐
Swam in salt water (sea) ☐ ☐ ☐ ☐ ☐
Swim in surface water (eg lake, river, but not in a swimming pool) ☐ ☐ ☐ ☐ ☐
Visited a sauna, hot tub or swimming pool ☐ ☐ ☐ ☐ ☐
Showed somewhere else than at home (for example at work, sports location, with family) ☐ ☐ ☐ ☐ ☐

Visited a car wash □ □ □ □ □
Worked with a high pressure cleaner, plant spray or garden hose □ □ □ □ □
Worked on the heating or water pipes? □ □ □ □ □
Visited a petting zoo □ □ □ □ □
Visited a playground visited □ □ □ □ □

PERMISSION STATEMENT

In order to be able to carry out the research, it is important that the following questions are answered.

25. In what age category do you / the addressee fall?

□ 0-12
□ 13-17
□ 18 and older

26. I want to be informed of the examination results and reports of the overall research results (not your personal results).

□ No.
□ Yes

27. I agree that the address details from this statement are used for follow-up investigation.

□ No.
□ Yes

28. Parent / caretaker 1: do you also agree with the above points, as entered by your child?

□ No.
□ Yes

29. Parent / caretaker 2: do you also agree with the above points, as entered by your child?

□ No.
□ Yes
□ Not applicable

30. Parent / caregiver 2: Do you also agree with the above points, as entered by your child's other parent / caregiver (if applicable)?

□ No.
□ Yes
□ Not applicable

Do you have any comments?

Thank you very much for your participation!

Supplement S3. Representativeness of the study population

		NL 2015	Provide feces	Difference
**Total**	**number**	16900726	4177	
**Age**				
**Younger than 20 years**	**%**	22.7	14.5	-8.2
**20 to 39 years**	**%**	24.5	12.5	-12
**40 to 64 years**	**%**	35.1	45	9.9
**65 to 79 years**	**%**	13.4	24.7	11.3
**80 years or older**	**%**	4.3	3.2	-1.1
**Mean age total population**	**year**	41.3	49.8	8.5
**Mean age males**	**year**	40.4	51.6	11.2
**Mean age females**	**year**	42.1	48.2	6.1
**Parents both born in the Netherlands**	**%**	78.3	97.5	19.2
**Males**	**%**	49.5	45.5	-4
**Females**	**%**	50.5	54.5	4
**Degree of urbanization (adresses per km2)**				
**>= 2500**	**%**	22.7	5.4	-17.3
**1500 - 2500**	**%**	24.8	15	-9.8
**1000 - 1500**	**%**	17.3	19.5	2.2
**500 - 1000**	**%**	17.6	27.9	10.3
**< 500**	**%**	17.6	32.2	14.6
**County**				
**Groningen**	**%**	3.5	4	0.5
**Friesland**	**%**	3.8	4.7	0.9
**Drenthe**	**%**	2.9	3.7	0.8
**Overijssel**	**%**	6.7	7.3	0.6
**Flevoland**	**%**	2.4	2	-0.4
**Gelderland**	**%**	12	15	3
**Utrecht**	**%**	7.5	6.5	-1
**Noord-Holland**	**%**	16.3	16.4	0.1
**Zuid-Holland**	**%**	21.3	14.6	-6.7
**Zeeland**	**%**	2.3	5	2.7
**Noord-Brabant**	**%**	14.7	13.6	-1.1
**Limburg**	**%**	6.6	7	0.4

The characteristics of the study population compared to the general Dutch population (2015) regarding age, ethnicity, gender, degree of urbanization and county.

Supplement S4. Descriptive statistics of all putative risk factors			
**Variable**	**Level**	**N=4,177 n**	**%**
A diet with beef	Yes	3831	97.6
	No	95	2.4
A diet with chicken meat	Yes	3877	96.9
	No	123	3.1
A diet with crustaceans	Yes	2019	53.2
	No	1774	46.8
A diet with dairy produce	Yes	4018	98.1
	No	76	1.9
A diet with eggs (well coocked)	Yes	3733	90.4
	No	396	9.6
A diet with fish	Yes	3830	94.0
	No	244	6.0
A diet with meat	Yes	3916	97.6
	No	95	2.4
A diet with pork	Yes	1577	80.7
	No	378	19.3
A Diet with raw eggs	Yes	265	7.3
	No	3347	92.7
A diet with shellfish	Yes	1469	39.2
	No	2278	60.8
Admission to hospital or long term care facility in the past 4 weeks	Yes	35	0.9
	No	3670	99.1
Age categories	0-4	180	4.3
	5-12	283	6.8
	13-19	143	3.4
	20-39	521	12.5
	40-64	1828	43.8
	65-79	1071	25.7
	80-Inf	149	3.6
Animal contact in the past 4 weeks	Yes	2143	58.3
	No	1534	41.7
Antibiotic use in the past	<6 months	450	11.4
	>12 months	2625	66.6
	6-12 months	304	7.7
	never	564	14.3
Antibiotic use in the past 8 weeks	Yes	262	7.0
	No	3456	93.0
Antidepressants use in the past 6 months	Yes	136	3.3
	No	3963	96.7
Antidiabetic use in the past 6 months	Yes	118	2.9
	No	3981	97.1
Antihypertensive drugs use in the past 6 months	Yes	736	18.0
	No	3363	82.0

Are hands washed after a toilet visit	Always	2546	62.4
	Not always	1534	37.6
Are hands washed before preparing food	Always	2505	60.7
	Not always	1622	39.3
Being abroad in the past 12 months	Yes	2813	68.3
	No	1306	31.7
Being abroad in the past 4 weeks	Yes	765	20.6
	No	2948	79.4
Children attending day-care in the household	Yes	733	22.0
	No	2600	78.0
Children in the household	Yes	1787	43.3
	No	2343	56.7
Children in the households wear diapers	Yes	379	11.4
	No	2957	88.6
Cholesterol-lowering medication used in the past 6 months	Yes	595	14.5
	No	3504	85.5
Chronic disease	Yes	1986	48.9
	No	2079	51.1
Cleaning the bathroom towel	Daily	659	18.4
	Not daily	2924	81.6
Cleaning the dishcloth	Daily	1560	38.5
	Not daily	2489	61.5
Cleaning the kitchen towel	Daily	970	23.6
	Not daily	3132	76.4
Contact with animal fur in the past 4 weeks	Yes	83	2.1
	No	3896	97.9
Contact with compost in the past 4 weeks	Yes	242	6.1
	No	3737	93.9
Contact with death animals in the past 4 weeks	Yes	141	3.8
	No	3591	96.2
Contact with death young animals in the past 4 weeks	Yes	46	1.2
	No	3686	98.8
Contact with hay and/or straw in the past 4 weeks	Yes	617	15.5
	No	3362	84.5
Contact with manure in the past 4 weeks	Yes	571	14.4
	No	3408	85.6
Contact with wool in the past 4 weeks	Yes	46	1.2
	No	3933	98.8
Country of birth	The Netherlands	4037	96.7
	Other	139	3.3
Degree of urbanization (addresses per km^2)	>= 2500	225	5.4
	1500 - 2500	625	15.0
	1000 - 1500	814	19.5
	500 - 1000	1166	27.9

	< 500	1346	32.2
Does someone of the household smoke	Yes	587	15.2
	No	3274	84.8
Eat in a restaurant	>20 times a year	431	10.4
	≤20 times a year	3698	89.6
Eat raw or undercooked meat one week prior to feacal sample collection	Yes	1189	32.2
	No	2509	67.8
Excrement of a rat seen in and around the house	Yes	559	13.6
	No	3558	86.4
Gender	Male	1899	45.5
	Female	2277	54.5
Had contact with someone with gastro-intestinal complaints in the past 4 weeks	Yes	603	19.2
	No	2537	80.8
Hospitalization in the past 12 months	Yes	334	8.1
	No	3798	91.9
Hospitalization of a household member in the past 12 months	Yes	404	11.1
	No	3245	88.9
Hospitalization of a household member in the past 4 weeks	Yes	164	4.4
	No	3547	95.6
How often is the toilet cleaned	≥once a week	2589	75.3
	<once a week	850	24.7
Keeping animals in the households	Yes	2016	49.1
	No	2089	50.9
Keeping farm animals	Yes	471	11.3
	No	3706	88.7
Keeping pets in the household	Yes	1766	43.0
	No	2339	57.0
Laxatives used in the past 6 months	Yes	162	4.0
	No	3937	96.0
Level of education	High	1532	37.5
	Intermediate	1917	46.9
	Low	637	15.6
Living along arable land	Yes	620	15.1
	No	3498	84.9
Living along open water	Yes	494	12.0
	No	3624	88.0
Living along the city park	Yes	451	11.0
	No	3667	89.0
Living along the forest edge	Yes	509	12.4
	No	3609	87.6
Living along the meadow	Yes	1062	25.8
	No	3056	74.2

Living in a long-term care facility	Yes	4	0.1
	No	4124	99.9
Occupational having contact with animals	Yes	95	2.3
	No	3953	97.7
Occupational having contact with children	Yes	321	8.0
	No	3707	92.0
Proton pump inhibitor used in the past 6 months	Yes	650	15.9
	No	3449	84.1
sedatives and tranquillizers used in the past 6 months	Yes	171	4.2
	No	3928	95.8
Socio-economic status (SES)	High	925	22.3
	Intermediate	2325	56.0
	Low	903	21.7
Stayed at the camping site in the past 12 months	Yes	1452	37.6
	No	2406	62.4
Stayed at the camping site in the past 4 weeks	Yes	248	7.9
	No	2884	92.1
Swimming in fresh water in the past 12 months	Yes	1038	27.7
	No	2713	72.3
Swimming in fresh water in the past 4 weeks	Yes	170	5.5
	No	2937	94.5
Swimming in sea/ocean in the past 12 months	Yes	1433	38.1
	No	2328	61.9
Swimming in sea/ocean in the past 4 weeks	Yes	184	5.9
	No	2930	94.1
The refrigerator is cleaned	≥monthly	1926	46.7
	<monthly	2201	53.3
Time between buying and storing chilled and/or deep frozen food in fridge and freezer at home	<15 minutes	1041	25.5
	15-29 minutes	2244	55.1
	>30 minutes	790	19.4
Use a clean/other knife for meat and vegetables	Yes	1669	41.0
	No	2406	59.0
Use of any medication	Yes	2557	62.4
	No	1542	37.6
Using the same cutting board for cutting meat and vegetable products	Yes	1765	42.8
	No	2290	55.5
	nvt	73	1.8
Was at a farm in the past 12 months	Yes	1193	32.0
	No	2535	68.0
Was at a farm in the past 4 weeks	Yes	318	10.2
	No	2803	89.8
Went to the forest in the past 12 months	Yes	3365	85.0
	No	594	15.0

Supplement S5. Descriptive statistics stratified by ESBL-E status

Variable	ESBL- (N=3991) n/N	ESBL- %	ESBL+ (N=186) n/N	ESBL+ %
**Gender (male)**	1805/3991	45.2	94/185	50.8
**Country of birth is other than the Netherlands**	3865/3991	96.8	172/185	93.0
**Age**				
0-4	173/3991	4.3	7/184	3.8
5-12	274/3991	6.9	9/184	4.9
13-19	136/3991	3.4	7/184	3.8
20-39	501/3991	12.6	20/184	10.9
40-64	1738/3991	43.5	90/184	48.9
65-79	1025/3991	25.7	46/184	25.0
>80	144/3991	3.6	5/184	2.7
**Social Economic Status (SES)**				
low	865/3970	21.8	38/183	20.8
intermediate	2231/3970	56.2	94/183	51.4
high	874 /3970	22.0	51/183	27.9
**Degree of urbanization**				
>= 2500	214/3991	5.4	11/185	5.9
1500 - 2500	591/3991	14.8	34/185	18.4
1000 - 1500	771/3991	19.3	43/185	23.2
500 - 1000	1120/3991	28.1	46/185	24.9
< 500	1295/3991	32.4	51/185	27.6
**Level of education**				
low	610/3908	15.6	27/178	15.2
intermediate	1850/3908	47.3	67/178	37.6
high	1448/3908	37.1	84/178	47.2
**Season**				
autumn	1242/3990	31.1	62/184	33.7
winter	987/3990	24.7	36/184	19.6
spring	835/3990	20.9	45/184	24.5
summer	926/3990	23.2	41/184	22.3
**Year**				
2014	346/3990	8.7	14/184	7.6
2015	1784/3990	44.7	67/184	36.4
2016	1860/3990	46.6	103/184	56.0
**Admission to hospital or long term care facility in the 4 weeks prior to fecal sample collection**	33/3543	0.9	2/162	1.2
**Used proton pump inhibitors in the past 6 months**	614/3919	15.7	36/180	20.0
**Ate in a restaurant more often than 20 times a year**	398/3946	10.1	33/183	18.0
**Not changing the kitchen towel on a daily basis**	2973/3920	75.8	159/182	87.4

**used antibiotics in the 8 weeks prior to fecal sample collection**	249/3556	7.0	13/162	8.0
**used antibiotics in the past**				
never	549/3773	14.6	15/170	8.8
<6 months	422/3773	11.2	28/170	16.5
6-12 months	291/3773	7.7	13/170	7.6
>12 months	2511/3773	66.6	114/170	67.1
**Traveling in the 4 weeks prior to fecal sample collection**	720/3551	20.3	45/162	27.8
**Attending day care of at least one child in the household**	243/3185	7.6	14/148	9.5
**Children <12 years old in the household**	966/3938	24.5	33/182	18.1
**Hospitalization in het past 12 months**	315/3950	8.0	19/182	10.4
**Animals in or around the household**	1914/3923	48.8	102/182	56.0
**Traveling abroad in the past 12 months**	2677/3937	68.0	136/182	74.7
**Traveling (in the past 12 months):**				
No	1260/3891	32.4	46/181	25.3
to Europe	2310/3891	59.4	100/181	55.2
To Africa	57/3891	1.5	8/181	4.4
to Asia	162/3891	4.2	22/181	12.2
to North-America	102/3891	2.6	5/181	2.8
**Swimming in sea/ocean in the past 12 months**	1353/3596	37.6	80/165	48.5
**Swimming in open fresh water in the past 12 months**	994/3590	27.7	44/161	27.3
**Swimming in sea/ocean in the past 4 weeks**	169/2979	5.7	15/135	11.1
**Swimming in open fresh water in the past 4 weeks**	158/2974	5.3	12/133	9.0
**Eat pork in the last week**	1506/1860	81.0	71/95	74.7
**Eat raw or undercooked meat in the week prior to fecal sample collection**	1134/3537	32.1	55/161	34.2
**Eat never meat**	3743/3825	97.6	173/177	97.7
**Living in a long term care facility**	4/3946	0.1	0/182	0.0

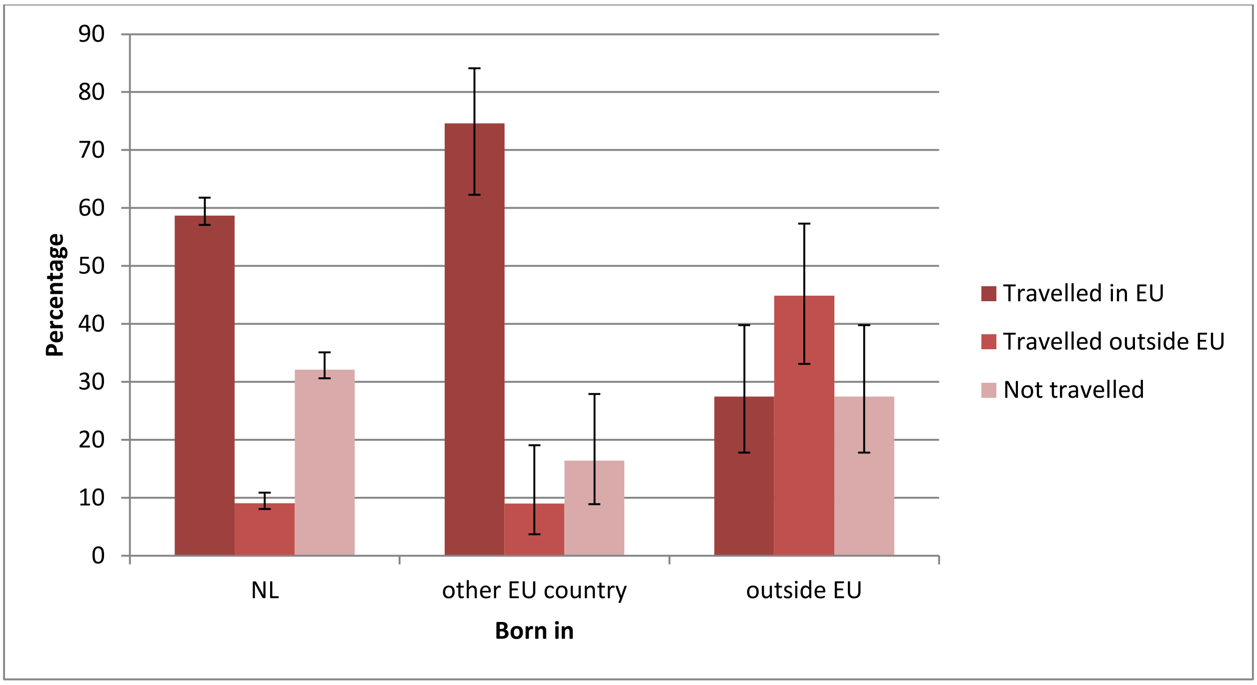

Supplement S6. Association between country of birth and travelling in the past 12 months
A higher percentage of participants born in another European country travels to a European country, and participants born outside Europe, to countries outside Europe. Overall, persons not born in the Netherlands are travelling more often compared to persons born in the Netherlands.

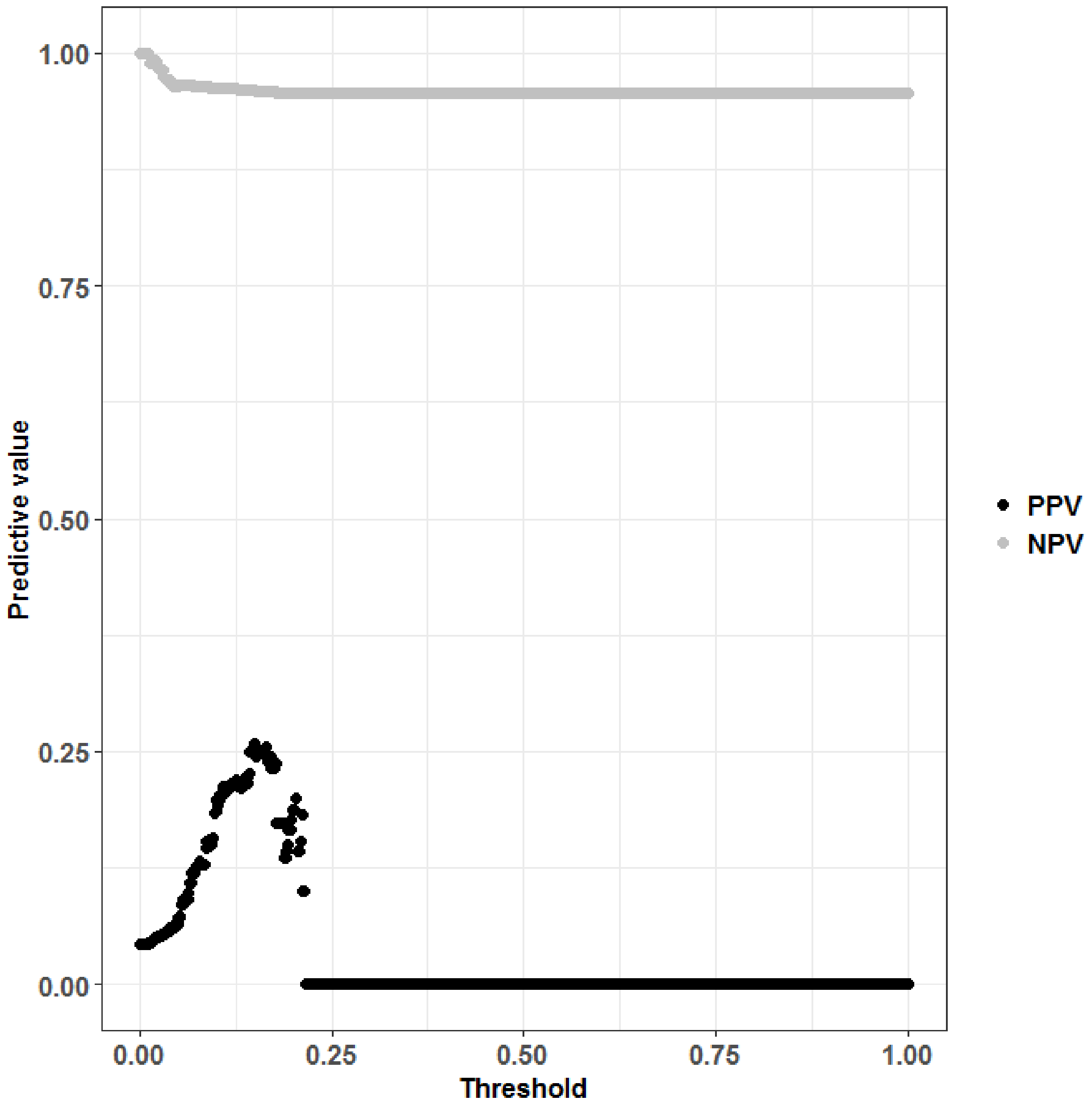

Supplement S7. Plot of the positive (PPV) and negative predictive value (NPV) for every possible predicted threshold.

By calculating the predicted probabilities of ESBL carriage based on the risk factors identified from the final multivariable logistic regression model, and subsequently calculating a PPV and NPV for every possible threshold between 0 and 1 with steps of .001, it appeared that the highest possible PPV is 0.26 and NPV is 1.
